# NMDA Receptors Regulate Oxidative Damage in Keratinocytes during Complex Regional Pain Syndrome in HaCaT Cells and Male Rats

**DOI:** 10.3390/antiox13020244

**Published:** 2024-02-18

**Authors:** Bei Wen, He Zhu, Jijun Xu, Li Xu, Yuguang Huang

**Affiliations:** 1 Department of Anesthesiology, Peking Union Medical College Hospital, Peking Union Medical College, Chinese Academy of Medical Sciences, Beijing 100730, China; pumc_wenbei@student.pumc.edu.cn (B.W.); zhuhe166959@student.pumc.edu.cn (H.Z.); 2 Department of Pain Management, Cleveland Clinic, Cleveland, OH 44195, USA; xuj3@ccf.org; 3 Department of Inflammation and Immunity, Cleveland Clinic, Cleveland, OH 44195, USA

**Keywords:** peripheral and central sensitization, dorsal root ganglion, chronic post-ischemia pain, differential expression analysis, ifenprodil, neuroinflammation, pain pathway, autonomic dysfunction, chronic pain

## Abstract

Complex regional pain syndrome (CRPS), a type of primary chronic pain, occurs following trauma or systemic disease and typically affects the limbs. CRPS-induced pain responses result in vascular, cutaneous, and autonomic nerve alterations, seriously impacting the quality of life of affected individuals. We previously identified the involvement of keratinocyte *N*-methyl-d-asparagic acid (NMDA) receptor subunit 2 B (NR2B) in both peripheral and central sensitizations in CRPS, although the mechanisms whereby NR2B functions following activation remain unclear. Using an in vivo male rat model of chronic post-ischemia pain (CPIP) and an in vitro oxygen–glucose deprivation/reoxygenation (OGD/R) cell model, we discovered that oxidative injury occurs in rat keratinocytes and HaCaT cells, resulting in reduced cell viability, mitochondrial damage, oxidative damage of nucleotides, and increased apoptosis. In HaCaT cells, OGD/R induced increases in intracellular reactive oxygen species levels and disrupted the balance between oxidation and antioxidation by regulating a series of antioxidant genes. The activation of NMDA receptors via NMDA exacerbated these changes, whereas the inhibition of the NR2B subunit alleviated them. Co-administration of ifenprodil (an NR2B antagonist) and NMDA (an NMDA receptor agonist) during the reoxygenation stage did not result in any significant alterations. Furthermore, intraplantar injection of ifenprodil effectively reversed the altered gene expression that was observed in male CPIP rats, thereby revealing the potential mechanisms underlying the therapeutic effects of peripheral ifenprodil administration in CRPS. Collectively, our findings indicate that keratinocytes undergo oxidative injury in CRPS, with NMDA receptors playing regulatory roles.

## 1. Introduction

Complex regional pain syndrome (CRPS) is a type of primary chronic pain that typically affects the limbs and can be classified as either CRPS type I or II based on the presence or absence of confirmed nerve injury, respectively [1]. CRPS invariable occurs as a consequence of trauma or systemic disease and presents as refractory pain accompanied by vascular, cutaneous, and autonomic nerve alterations. These changes result in substantial functional impairment and disability, which can have a significant effect on daily life [2,3]. Furthermore, it is worth noting that acute stressors, such as significant nociceptive stimuli and surgical procedures, can elicit a hyperinflammatory response and potentially trigger the development of CRPS following trauma [4]. Although the mechanisms of CRPS are complex and remain poorly understood, potential mechanisms are believed to involve peripheral and central sensitization, systemic inflammation, autonomic dysfunction, and neuroinflammation [5,6,7,8,9]. Given that the current treatment options for CRPS are limited, a more comprehensive understanding of the underlying mechanisms would make a significant contribution toward the development of novel therapeutic strategies.

The chronicity of pain is dependent on peripheral sensitization, which refers to a reduction in the nociceptive threshold and augmented sensitivity to painful stimuli due to persistent nociceptive stimulation (e.g., tissue damage or inflammation) [10,11]. The skin serves as the body’s primary interface with the external environment and plays a pivotal role in defense and sensory perception. Keratinocytes are the predominant cell type within the epidermis and make a significant contribution to peripheral pain sensitization [12]. Using a chronic post-ischemia pain (CPIP) model to mimic CRPS in male rats, our team previously identified the involvement of keratinocytes and *N*-methyl-d-asparagic acid (NMDA) receptor subunit 2 B (NR2B) in both the peripheral and central sensitization of CRPS [13]. However, the mechanisms whereby NMDA receptors function following activation have yet to be determined.

Oxidative stress and chronic inflammation have been established to be significant contributors to the development of chronic pain [14]. Important manifestations of CRPS include disturbance of the microcirculation and tissue hypoxia [15], and it has previously been shown that local oxidative injury occurs in CRPS and sensitizes the nociceptors [16,17]. Furthermore, NR2B inhibitors have been found to reverse injuries that are caused by oxygen–glucose deprivation (OGD) in the central nervous system and alleviate oxidative injury in rats with brain ischemia/reperfusion (I/R) injury [18,19].

On the basis of our current understanding of CRPS, we hypothesized that keratinocytes would undergo oxidative damage in CRPS and that peripheral NMDA receptors are involved in regulating this process and modulating gene expression profiles in the dorsal root ganglion (DRG). To verify this hypothesis, we established an oxygen–glucose deprivation/reoxygenation (OGD/R) cell model and a male CPIP rat model to elucidate the mechanisms underlying the activation of keratinocyte NMDA receptors in CRPS.

## 2. Materials and Methods

### 2.1. Cell Culture and Establishment of an OGD/R HaCaT Cell Model

The HaCaT human keratinocyte cell line used in this study was obtained from China Infrastructure of Cell Line Resource (Beijing, China). These cells were cultured in Minimum Essential Medium (MEM) α (Cat No: 112571063; Gibco, New York, NY, USA), supplemented with 10% fetal bovine serum (Cat No: 16000044;Gibco, New York, NY, USA) and 1% penicillin–streptomycin (Cat No: 15140-122;Gibco, New York, NY, USA) at a temperature of 37 °C under a controlled atmosphere of 5% CO_2_ and saturated humidity. HaCaT cells were subjected to OGD/R to induce CRPS in vitro. Glucose-free MEM (Cat No: PM150443; Pricella, Wuhan, China) was used to induce OGD for 2 h under low-oxygen conditions, followed by replacement with complete medium in normal condition for 24 h.

### 2.2. In Vitro Experimental Design and Grouping

To determine whether keratinocyte NMDA receptors influence OGD/R-induced oxidative injury in HaCaT cells, we used three drugs in this study: *N*-acetyl-l-cysteine (NAC: an antioxidant, Cat No: S0077; Beyotime, Shanghai, China), NMDA (an agonist for NMDA receptor, Cat No: B1624; APExBIO Technology, Houston, TX, USA), and ifenprodil (a selective NR2B antagonist, Cat No: B1623; APExBIO Technology, Houston, TX, USA). The HaCaT cells were divided into the following six groups: Control, OGD/R, OGD/R+NAC (10 mM NAC was used during the reoxygenation process), OGD/R+I (10 μM ifenprodil was used during the reoxygenation process), OGD/R+N (200 μM NMDA was used during the reoxygenation process), and OGD/R+N+I (200 μM NMDA and 10 μM ifenprodil were used during the reoxygenation process). Dosages were determined based on those previously reported [13,20,21,22,23].

### 2.3. Animals and the Rat Model of CPIP

Experimental procedures involving animals adhered to the guidelines set forth by the Committee for Research and Ethical Issues of the International Association of Pain (IASP) and underwent welfare and ethical inspection conducted by the Peking Union Medical College Hospital (PUMCH) Animal Welfare and Ethics Committee (XHDW-2022-043). Animal research was reported in adherence with the ARRIVE guidelines 2.0 https://arriveguidelines.org/resources/author-checklists (accessed on 29 January 2024). Adult male Sprague–Dawley rats (procured from BEIJING HFK BIOSCIENCE CO., LTD, Beijing, China) were housed in the Experimental Animal Center of PUMCH (Beijing, China) under standard conditions (3 rats per cage, under a 12 h/12 h light/dark cycle, at an ambient temperature of 25 ± 3 °C), with ad libitum access to food and water. The rats in all experimental groups were subjected to identical environmental conditions.

The CPIP rat model used in this study was previously developed and demonstrated to successfully replicate the characteristics of CRPS, and it is widely employed for studying CRPS [17,24,25,26,27]. Given the influence of hormone circulation and estrus in female rats [13,17], male rats were used exclusively in this study. Rats weighing 200–220 g were anesthetized via intraperitoneal injection of 60 mg/kg sodium pentobarbital, followed by 20 mg/kg injections at 60 min intervals, depending on the response to anesthesia. A Nitrile 70 Durometer O-ring with an internal diameter of 0.56 cm (Kangda Chemical Company, Shanghai, China) was tightly placed around the right hind limb of rats proximal to their medial malleolus and was kept on the limb for 3 h, prior to being cut to enable blood reperfusion before emergence from anesthesia. The ankle joints of sham rats were loosely surrounded by cut O-rings.

### 2.4. Animal Study Design

The protocol was designed before experiments. Rats were allocated to sham and CPIP groups in a randomized manner using tables of random numbers. The CPIP group was further divided into the following four treatment subgroups: CPIP alone, CPIP plus 100 μL normal saline (NS), CPIP plus 100 μL of 220 mM NMDA, and CPIP plus 100 μL of 1 mM ifenprodil. Sample sizes were calculated by Resource Equation Approach for group comparison—one-way ANOVA (n = degrees of freedom/group number + 1) [28]. The minimum sample size required for each group was 3. Within each group, 3 rats were subjected to immunofluorescence testing, while the other 3 rats were used for transmission electron microscopy and RNA-seq analysis. In conclusion, a total of 30 rats were utilized in this study, with 6 rats allocated to each group, all of which underwent behavioral tests. From days 8 to 14 after modeling, the drugs were subcutaneously injected once daily into the ventral side of the hind paw on the I/R side. Behavioral assessments were conducted at baseline and on days 7, 10, and 14 post-CPIP induction. Tissues were harvested on day 14. The timeline of the animal experiments is shown in Supplementary Figure S1. No animal was excluded for analysis.

### 2.5. Behavioral Tests

All behavioral tests were conducted by an investigator who was blinded to the experimental design and drug administration. Mechanical allodynia and thermal hyperalgesia were used to evaluate nociceptive behavior. Prior to testing, rats were acclimated to the testing environment for 30 min daily for three consecutive days and were acclimated to the measurement environment for 30 min prior to each behavioral test. Hind paw mechanical allodynia was assessed using a calibrated electronic von Frey apparatus (IITC, Los Angeles, CA, USA). Simultaneously, hind paw thermal hyperalgesia was evaluated using a radiant heat device (Cat No: BME-410A; Chinese Institute of Biomedical Engineering, China) focused on the plantar surface of the ipsilateral hind paw, with repeated measurements of hind paw withdrawal threshold and latency taken three times at approximately 10 min intervals, with the values thus obtained being averaged.

### 2.6. Cell Viability Assay and Tunnel Staining

Cell viability and cytotoxicity were assessed using propidium iodide (PI) and calcein acetoxymethyl ester (calcein-AM) staining, following the instructions of a Calcein/PI Cell Viability/Cytotoxicity Assay Kit (Cat No: C2015S; Beyotime, Shanghai, China). Levels of apoptosis were evaluated using a terminal deoxynucleotidyl transferase-mediated dUTP-biotin nick-end labeling (TUNEL) assay using a one-step TUNEL kit (Cat No: C1090; Beyotime, Shanghai, China) according to the manufacturer’s instructions. An Axio Imager Z2 fluorescence microscope (Zeiss, Oberkochen, Germany) was used for imaging.

### 2.7. Immunofluorescence

Cells were cultured on ϕ24 glass coverslips (Cat No: AQ53006; Beijing Aoqing Biotechnology Co., Ltd., Beijing, China). Rats were anesthetized with an intraperitoneal injection of 40 mg/kg sodium pentobarbital and perfused with saline, followed by fresh 4% paraformaldehyde (PFA). The plantar skin of the ipsilateral hind paw was harvested, fixed in 4% PFA at 4 °C for 24 h, and sequentially subjected to dehydration using 20% and 30% sucrose solutions. Samples were then embedded in optimal cutting temperature compound (Sakura Finetek, Tokyo, Japan), frozen at −20 °C for 2 h, cut into 8 μm thick frozen sections, mounted on Superfrost Plus microscope slides (Shitai, Jiangsu, China), and stored at −80 °C. The cell coverslips and skin sections were fixed with 4% PFA for 30 min and permeabilized with 0.3% Triton X-100 for 10 min at room temperature (approx. 30 °C). Thereafter, the sections were blocked with 5% normal goat serum at room temperature for 1 h and incubated with primary antibodies (Supplementary Table S1) in a wet box overnight at 4 °C. The following morning, the appropriate secondary antibodies were added for 2 h after washing. The cells were washed three times with PBS and sealed with an antifade solution containing DAPI. Finally, the cell coverslips and skin sections were examined using an Axio Imager Z2 fluorescence microscope (Zeiss, Germany). The intensity of immunofluorescence was analyzed using ImageJ software (National Institutes of Health, Bethesda, MD, USA).

### 2.8. Measurement of Mitochondrial Membrane Potential

Changes in the mitochondrial membrane potential (MMP) were assessed using a JC-1 assay kit (C2006; Beyotime, Shanghai, China) according to the manufacturer’s instructions. Briefly, HaCaT cells were seeded onto 8 well-chambered cover glasses (Cat No: C8-1.5H-N; Cellvis, Sunnyvale, CA, USA) and treated as previously described in Section 2.2. Subsequently, 1× JC-1 dye was added to each well for 30 min, followed by two washes with the dilution buffer. Finally, complete medium was added to the wells, and cellular observations were performed using an LSM900 laser scanning confocal microscope (Zeiss, Germany). As an indicator of MMP, we assessed the ratio of the JC-1 monomer fluorescence intensity to that of aggregates.

### 2.9. Transmission Electron Microscopy

After digestion with trypsin, HaCaT cells were centrifuged at 1000 rpm for 5 min, and the resulting supernatant was discarded. Fresh rat skin tissues were sliced into strips less than 1 mm wide. The cells and skin sections were fixed overnight at 4 °C in 2.5% EM-grade glutaraldehyde (P1126; Solarbio Life Sciences, Beijing, China), followed by fixation in 1% osmic acid. Thereafter, the sections were dehydrated and embedded using standard procedures. Ultrathin sections (0.08 μm) were stained with lead citrate and uranyl acetate for approximately 5–10 min at a temperature of approximately 95 °C, after which, appropriate areas were selected for transmission electron microscopy (TEM) analysis using a JEM-1400Plus transmission electron microscope (JEOL Ltd., Tokyo, Japan).

### 2.10. Real-Time Quantitative Polymerase Chain Reaction

Total RNA was extracted from HaCaT cells using an RNA extraction kit (Cat No: B0132; HaiGene, Harbin, China) following the manufacturer’s instructions. The extracted RNA was qualified and reverse-transcribed using Prime Script RT Master Mix (RR036; Takara, Otsu, Japan). Quantitative polymerase chain reactions (qPCR) were performed using SYBR Premix Ex Taq (Cat No: RR820, Takara, Osaka, Japan) and a StepOne Real-Time PCR System (Applied Biosystems, Foster City, CA, USA), with each sample being assessed in three replicate wells. The primers used for qPCR are listed in Supplementary Table S2.

### 2.11. RNA-Seq Analysis

For RNA-seq analysis, we utilized Lumbar (L) 3-5 DRG in this study, as the sensory innervation of the posterior limbs’ skin in rats was primarily provided by the sciatic and saphenous nerves, which predominantly originate from L3-L5 DRGs [29]. Total RNA was extracted from DRGs using an RNA extraction kit (B0132; HaiGene, China) following the manufacturer’s protocols. Strand-specific libraries were constructed using a TruSeq RNA sample preparation kit (Illumina, San Diego, CA, USA), and sequencing was performed using an Illumina Novaseq 6000 instrument. Skewer was used to process the raw data, whereas FastQC v0.11.2 ensured the selection of high-quality datasets for transcript quantification, measured as FPKM values using Perl scripts. Genes that were differentially expressed (DEGs) between groups were identified using an MA-plot-based method with a Random Sampling model in the DEGseq package. Functional and signaling pathway enrichment analyses of the DEGs were conducted using the Gene Ontology (GO) and Kyoto Encyclopedia of Genes and Genomes (KEGG) databases. Identification of significantly enriched pathways was based on a *p* < 0.05 and the inclusion of at least two affiliated genes. For Gene Set Enrichment Analysis (GSEA), genes were ranked according to their degree of differential expression in the two samples prior to assessing predefined gene sets for enrichment at either end of this list, while independently using a local version of the GSEA analysis tool (http://www.broadinstitute.org/gsea/index.jsp, accessed on 6 June 2023) with the Hallmark dataset.

### 2.12. Statistical Analysis

Quantitative variables are presented as the means ± standard deviation. Student’s *t*-test was used to compare differences between two groups, whereas repeated measures one-way ANOVAs were used for comparisons among three or more groups. Post hoc tests with Bonferroni correction were applied for multiple comparisons. Statistical significance was set at *p* < 0.05. All statistical analyses were performed using GraphPad Prism 8.0.2 (GraphPad Software, Inc., San Diego, CA, USA).

## 3. Results

### 3.1. Keratinocytes in Male Rats with CPIP Show Oxidative Damage

**Figure 1:**
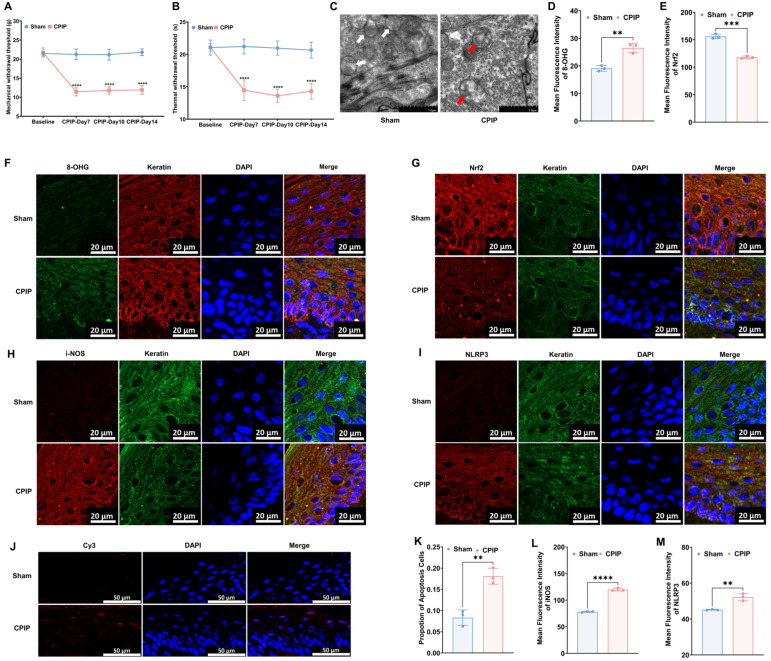
Keratinocytes in male rats with CPIP experience oxidative injury. (**A**) Mechanical allodynia in rats with CPIP (*n* = 6). (**B**) Thermal allodynia in rats with CPIP (*n* = 6). (**C**) Transmission electron microscopy shows that the hind paw skin of rats with CPIP exhibited mitochondrial morphological changes. White arrows represent normal mitochondria (characterized by oval or round shape with regularly arranged mitochondrial cristae in tubules), while red arrows denote impaired mitochondria (exhibiting mitochondrial swelling, vacuolar degeneration, and disordered cristae structure). (**D**) Quantitative analysis of the 8-OHG immunofluorescence intensity from (**F**) (*n* = 3). (**E**) Quantitative analysis of the Nrf2 immunofluorescence intensity from (**G**) (*n* = 3). (**F**) Immunofluorescence reveals an increase in 8-OHG levels in the keratinocytes of rats with CPIP. (**G**) Immunofluorescence reveals downregulation of Nrf2 in the keratinocytes of rats with CPIP. (**H**) Immunofluorescence shows upregulation of iNOS in the keratinocytes of rats with CPIP. (**I**) Immunofluorescence reveals upregulation of NLRP3 in the keratinocytes of rats with CPIP. (**J**) Tunnel staining revealed an increase in keratinocyte apoptosis among rats with CPIP. (**K**) Quantitative analysis of apoptotic cell number from (**J**) (*n* = 3). (**L**) Quantitative analysis of iNOS immunofluorescence intensity from (**H**) (*n* = 3). (**M**) Quantitative analysis of NLRP3 immunofluorescence intensity from (**I**) (*n* = 3). **, *p* < 0.01; ***, *p* < 0.005; ****, *p* < 0.001. TEM, transmission electron microscopy; CPIP, chronic post-ischemia pain; 8-OHG, 8-hydroxy-2 deoxyguanosine; NLRP3, NOD-like receptor thermal protein domain associated protein 3; Nrf-2, nuclear factor erythroid 2-related factor 2; iNOS, inducible nitric oxide synthase; Cy3, cyanine 3.

To investigate the occurrence of oxidative injury in keratinocytes in CRPS, we established a rat model to mimic CRPS in vivo. As depicted in Figure 1, rats with CPIP were characterized by significant mechanical and thermal allodynia, thereby indicating the successful induction of CRPS. To examine different oxidative and antioxidative targets, we collected hairless skin from the hind paws of the affected limbs. The TEM analysis revealed mitochondrial impairment in the keratinocytes of rats with CPIP (Figure 1C), whereas the immunofluorescence analysis revealed an increase in the level of 8-OHG (a marker of DNA/RNA damage caused by oxidation) in the keratin-positive cells of rats with CPIP (Figure 1D,F; CPIP vs. sham, *p* < 0.01). We also observed a downregulation of Nrf2, a key antioxidant factor, in the keratinocytes of rats with CPIP (Figure 1E,G; CPIP vs. sham, *p* < 0.005). Moreover, we found that the keratinocytes in these rats expressed high levels of i-NOS (Figure 1H,L; CPIP vs. sham, *p* < 0.001) and the NLRP3 inflammasome (Figure 1I,M; CPIP vs. sham, *p* < 0.01). To examine the apoptosis of keratinocyte in the affected hind paw, we performed a tunnel test, which compared with the sham group, revealed a higher rate of apoptosis in rats with CPIP (*p* < 0.01; Figure 1J,K).

### 3.2. OGD/R Induces Oxidative Damage in HaCaT Cells

**Figure 2:**
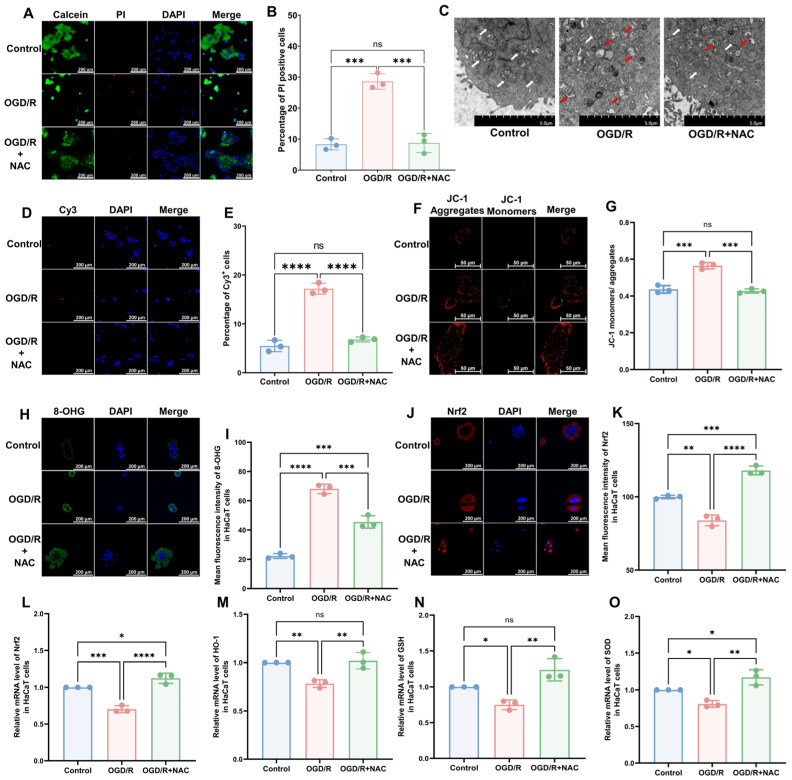
HaCaT cells exhibit oxidative damage after OGD/R, which can be reversed by antioxidants. (**A**) Cell viability and cytotoxicity assays indicated a reduction in cell viability after OGD/R, which was reversed by NAC treatment. (**B**) Quantitative analysis of PI-positive cells in (**A**) (*n* = 3). (**C**) TEM revealed that HaCaT cells exhibit changes in mitochondrial morphology during OGD/R, which were ameliorated by NAC. White arrows represent normal mitochondria (characterized by oval or round shape with regularly arranged mitochondrial cristae in tubules), while red arrows denote impaired mitochondria (exhibiting mitochondrial swelling, vacuolar degeneration, and disordered cristae structure). (**D**) Tunnel staining analysis revealed that OGD/R treatment induces apoptosis in HaCaT cells, which was ameliorated by NAC. (**E**) Quantitative analysis of Cy3-positive cells in (**D**) (*n* = 3). (**F**) JC-1 assay revealed a reduction in mitochondrial potential in OGD/R HaCaT cells, which was ameliorated upon treatment with NAC. (**G**) Quantitative analysis of the JC-1 monomer-to-aggregate ratio in (**F**) (*n* = 3). (**H**) Immunofluorescence revealed an increase in DNA/RNA damage in OGD/R HaCaT cells, which was mitigated by NAC. (**I**) Quantitative analysis of 8-OHG immunofluorescence intensity in (**H**). (**J**) Immunofluorescence shows that Nrf2 was downregulated by OGD/R and upregulated by NAC. (**K**) Quantitative analysis of Nrf2 immunofluorescence intensity in (**J**). (**L**–**O**) Quantitative real-time PCR revealed changes in mRNA levels of Nrf2 (**L**), HO-1 (**M**), GSH (**N**), and SOD (**O**) following OGD/R and NAC treatment (*n* = 3). ns, no significance; *, *p* < 0.05; **, *p* < 0.01; ***, *p* < 0.005; ****, *p* < 0.001. Cy3, cyanine 3; PI, propidium iodide; NAC, *N*-acetyl-l-cysteine; OGD/R, oxygen–glucose deprivation/reoxygenation; TEM, transmission electron microscopy; 8-OHG, 8-hydroxy-2 deoxyguanosine; Nrf-2, nuclear factor erythroid 2-related factor 2; HO-1, heme oxygenase-1; GSH, glutathione; SOD, superoxide dismutase.

To model CRPS in vitro, we selected the human immortalized keratinocyte HaCaT cell line and induced I/R injury using OGD/R. The results of a cell viability assay revealed that OGD/R increased the proportion of deceased cells, which was subsequently reversed by the administration of the antioxidant NAC (Figure 2A,B; OGD/R vs. Control, *p* < 0.005; OGD/R+NAC vs. OGD/R, *p* < 0.005; OGD/R+NAC vs. Control, *p* > 0.05). Similar to the in vivo model, we found that OGD/R induced mitochondrial damage (Figure 2C), a higher rate of apoptosis (Figure 2D,E; OGD/R vs. Control, *p* < 0.005; OGD/R+NAC vs. OGD/R, *p* < 0.005; OGD/R+NAC vs. Control, *p* > 0.05), higher 8-OHG levels (Figure 2H,I; OGD/R vs. Control, *p* < 0.001; OGD/R+NAC vs. OGD/R, *p* ˂ 0.005; OGD/R+NAC vs. Control, *p* ˂ 0.005), and lower levels of Nrf2 (Figure 2J,K; OGD/R vs. Control, *p* ˂ 0.01; OGD/R+NAC vs. OGD/R, *p* ˂ 0.001; OGD/R+NAC vs. Control, *p* ˂ 0.005) in HaCaT cells. Moreover, we found that NAC can reverse all these changes. The findings of JC-1 and reactive oxygen species (ROS) assays revealed that OGD/R reduced the mitochondrial membrane potential (Figure 2F,G) and elevated intracellular ROS levels (Supplementary Figure S2A,B), whereas treatment with NAC alleviated these injuries. Consistently, RT-qPCR revealed a significant reduction in the mRNA expression levels of Nrf2 (Figure 2L) and its downstream targets HO-1 (Figure 2M), GSH (Figure 2N), and SOD (Figure 2O) following OGD/R and OGD/R+NAC treatment.

### 3.3. NMDA Receptors Modulate Oxidative Damage in Keratinocytes of CRPS

**Figure 3:**
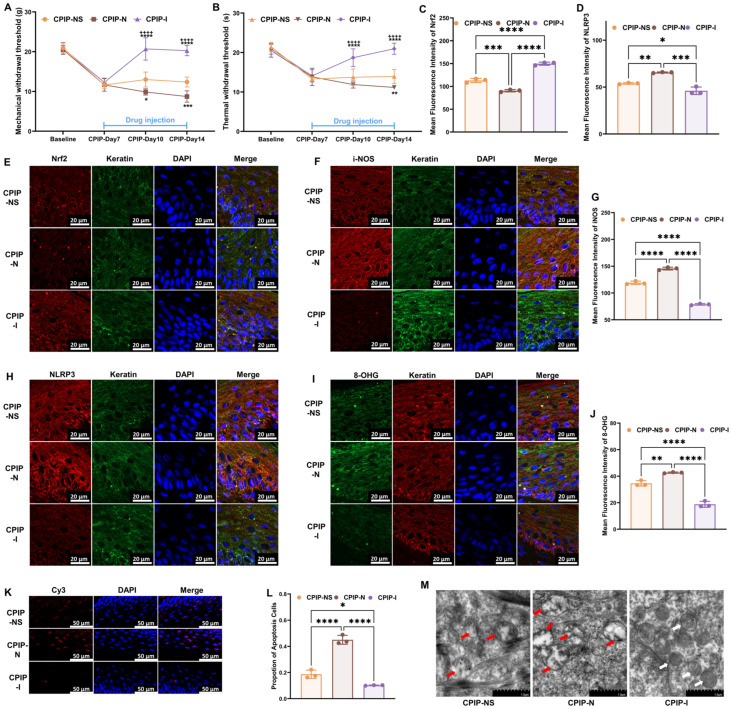
NMDA receptors modulate oxidative damage in keratinocytes of male rats with CPIP. (**A**) NMDA receptors regulate mechanical allodynia in rats with CPIP (*n* = 6). (**B**) NMDA receptors regulate thermal allodynia in rats with CPIP (*n* = 6). (**C**) Quantitative analysis of Nrf2 immunofluorescence intensity in (**E**) (*n* = 3). (**D**) Quantitative analysis of NLRP3 immunofluorescence intensity in (**H**) (*n* = 3). (**E**,**F**,**H**) Immunofluorescence reveals that NMDA receptors modulate the expression of Nrf2 (**E**), i-NOS (**F**), and NLRP3 (**H**) in keratinocytes from rats with CPIP. (**G**) Quantitative analysis of iNOS immunofluorescence intensity in (**F**) (*n* = 3). (**I**) Immunofluorescence indicates that NMDA receptors modulate the DNA/RNA damage in keratinocytes of rats with CPIP. (**J**) Quantitative analysis of i-NOS immunofluorescence intensity in (**I**) (*n* = 3). (**K**) Tunnel staining revealed that NMDA receptors regulate keratinocytes apoptosis among rats with CPIP. (**L**) Quantitative analysis of apoptotic cell number from (**K**) (*n* = 3). (**M**) TEM shows that NMDA receptors alter mitochondrial damage in the keratinocytes of male rats with CPIP. White arrows represent normal mitochondria (characterized by oval or round shape with regularly arranged mitochondrial cristae in tubules), while red arrows denote impaired mitochondria (exhibiting mitochondrial swelling, vacuolar degeneration, and disordered cristae structure). *, compared with sham, *p* < 0.05; **, compared with sham, *p* < 0.01; ***, compared with sham, *p* < 0.005; ****, compared with sham, *p* < 0.001; ^++++^, compared with CPIP-N, *p* < 0.001. TEM, transmission electron microscopy; CPIP, chronic post-ischemia pain; 8-OHG, 8-hydroxy-2 deoxyguanosine; NLRP3, NOD-like receptor thermal protein domain associated protein 3; Nrf-2, nuclear factor erythroid 2-related factor 2; iNOS, inducible nitric oxide synthase; Cy3, cyanine 3.

To investigate the potential regulatory role of NMDA receptors in the oxidative damage of keratinocytes under CRPS both in vivo and in vitro, we further examined oxidation-related targets following treatment with NMDA or ifenprodil, based on our previous findings. Initially, from days 8 to 14, NMDA or ifenprodil was administered daily via intraplanar injection in male rats with CPIP (Supplementary Figure S1). As shown in Figure 3, NMDA exacerbated the damage caused by CPIP in keratinocytes, whereas ifenprodil alleviated the CPIP-induced injuries. These injuries included mechanical (Figure 3A) and thermal (Figure 3B) allodynia, Nrf2 levels in keratinocytes (Figure 3C,E), i-NOS levels in keratinocytes (Figure 3F,G), NLRP3 levels in keratinocytes (Figure 3D,H), 8-OHG levels in keratinocytes (Figure 3I,J), the proportion of apoptotic keratinocytes (Figure 3K,L), and mitochondrial morphology in keratinocytes (Figure 3M).

**Figure 4:**
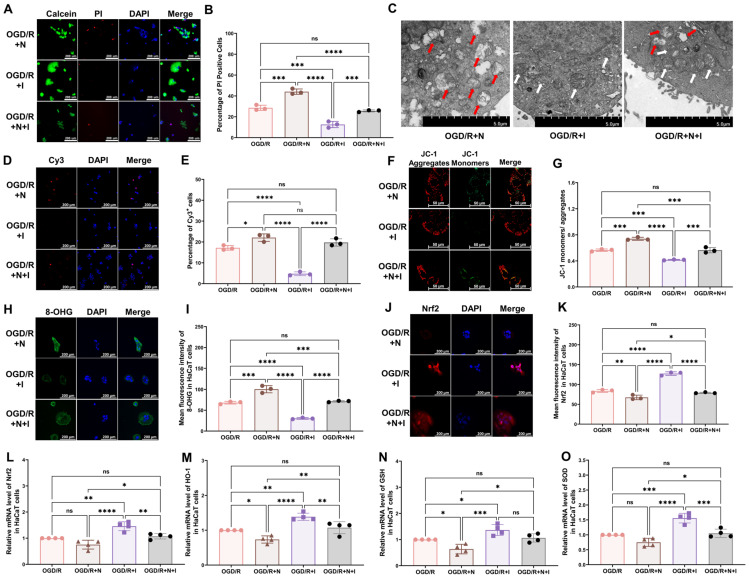
NMDA receptors modulate OGD/R-induced oxidative damage in HaCaT cells. (**A**) Cell viability and cytotoxicity assays indicated that NMDA receptors modulate the viability of OGD/R HaCaT cells. (**B**) Quantitative analysis of PI-positive cells in (**A**) (*n* = 3). (**C**) TEM revealed that NMDA receptors regulate changes in mitochondrial morphology in OGD/R HaCaT cells. White arrows represent normal mitochondria (characterized by oval or round shape with regularly arranged mitochondrial cristae in tubules), while red arrows denote impaired mitochondria (exhibiting mitochondrial swelling, vacuolar degeneration, and disordered cristae structure). (**D**) Tunnel staining revealed that NMDA receptors regulate apoptosis in OGD/R HaCaT cells. (**E**) Quantitative analysis of Cy3-positive cells in (**D**) (*n* = 3). (**F**) JC-1 assay revealed that NMDA receptors modulate the mitochondrial potential in OGD/R HaCaT cells. (**G**) Quantitative analysis of the JC-1 monomer-to-aggregate ratio in (**F**) (*n* = 3). (**H**) Immunofluorescence reveals that NMDA receptors regulate DNA/RNA damage in OGD/R HaCaT cells. (**I**) Quantitative analysis of 8-OHG immunofluorescence intensity in (**H**) (*n* = 3). (**J**) Immunofluorescence shows that NMDA receptors regulate Nrf2 expression in OGD/R HaCaT cells. (**K**) Quantitative analysis of Nrf2 immunofluorescence intensity in (**J**) (*n* = 3). (**L**–**O**) Quantitative real-time PCR revealed that NMDA receptors regulate changes in the mRNA levels of Nrf2 (**L**), HO-1 (**M**), GSH (**N**), and SOD (**O**) in OGD/R HaCaT cells (*n* = 4). ns, no significance; *, *p* < 0.05; **, *p* < 0.01; ***, *p* < 0.005; ****, *p* < 0.001. N, NMDA group; I, ifenprodil group; Cy3, cyanine 3; PI, propidium iodide; OGD/R, oxygen–glucose deprivation/reoxygenation; NMDA, N-methyl-D-aspartate; TEM, transmission electron microscopy; 8-OHG, 8-hydroxy-2 deoxyguanosine; Nrf-2, nuclear factor erythroid 2-related factor 2; HO-1, heme oxygenase-1; GSH, glutathione; SOD, superoxide dismutase.

To assess the effect of NMDA receptors on oxidative injury in keratinocytes in vitro, NMDA, ifenprodil, or both was added to the culture medium at the onset of reoxygenation. Ifenprodil was found to alleviate OGD/R-induced changes, including the reduced cell viability (Figure 4A,B), damaged mitochondria (Figure 4C), increased cell apoptosis (Figure 4D,E), and reduced mitochondrial potential (Figure 4F,G), and to promote an elevation in the levels of 8-OHG (Figure 4H,I) and intracellular ROS (Supplementary Figure S2C,D), reduce Nrf2 levels (Figure 4J,K), and reduce the mRNA levels of antioxidative proteins (Figure 4L–O: Nrf2, HO-1, GSH, SOD). Moreover, NMDA was found to exacerbate all OGD/R-induced changes in Figure 2. To further explore the role of NMDA receptors, NMDA and ifenprodil were simultaneously administered in the reoxygenation stage. Interestingly, no obvious changes were found compared with the OGD/R group, which indicates that the NR2B subunit might be the main subunit in this process.

### 3.4. NR2B Induces Changes in the Gene Transcriptome Profiles of DRGs in Male Rats with CPIP

**Figure 5:**
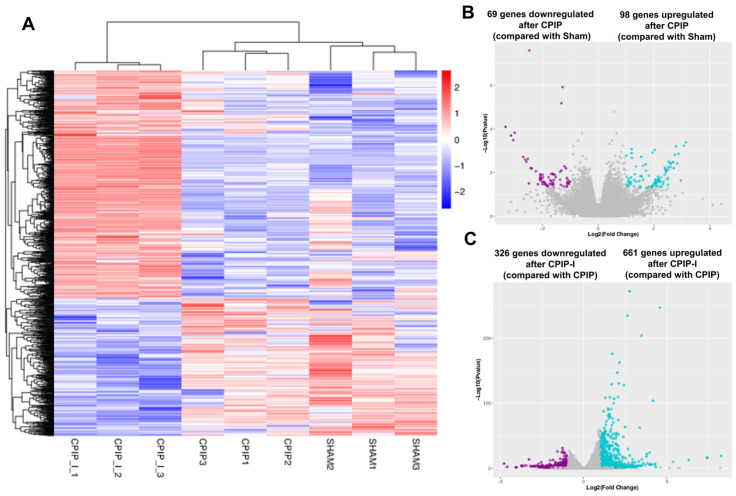
RNA-seq reveals alterations in the transcriptome profiles of gene expression in the DRGs of male rats induced by CPIP and intraplantar injection of ifenprodil. (**A**) A heat map of the hierarchical clustering of DEGs among sham, CPIP, and CPIP+I groups. (**B**) A Volcano plot shows the differential gene expression patterns observed in DRGs of sham and CPIP groups. (**C**) A Volcano plot shows the DEG patterns observed in the DRGs of CPIP-I and CPIP groups. RNA-Seq, RNA sequencing; DRG, dorsal root ganglion; CPIP, chronic post-ischemia pain; DEGs: differentially expressed genes; I, ifenprodil.

The impacts of CPIP and intraplantar ifenprodil injection on the gene transcriptome profiles of DRGs in male rats were investigated using mRNA sequencing, with more than 95% of the clean reads in each sample being successfully mapped to the rat genome (Supplementary Table S3). Figure 5A shows that samples from the sham, CPIP, and CPIP-I groups clustered into distinct groups, indicating a clear segregation between, although not within, different groups. Compared with the sham group, we identified a total of 167 DEGs following the induction of CPIP, of which 69 and 98 were down- and upregulated, respectively (Figure 5B). In contrast, 987 DEGs were identified in the CPIP-I vs. CPIP groups, of which 326 and 661 were down- and upregulated, respectively (Figure 5C). Detailed information on the top 20 up- and downregulated differential genes between CPIP-I and CPIP is presented in Supplementary Tables S4 and S5.

**Figure 6:**
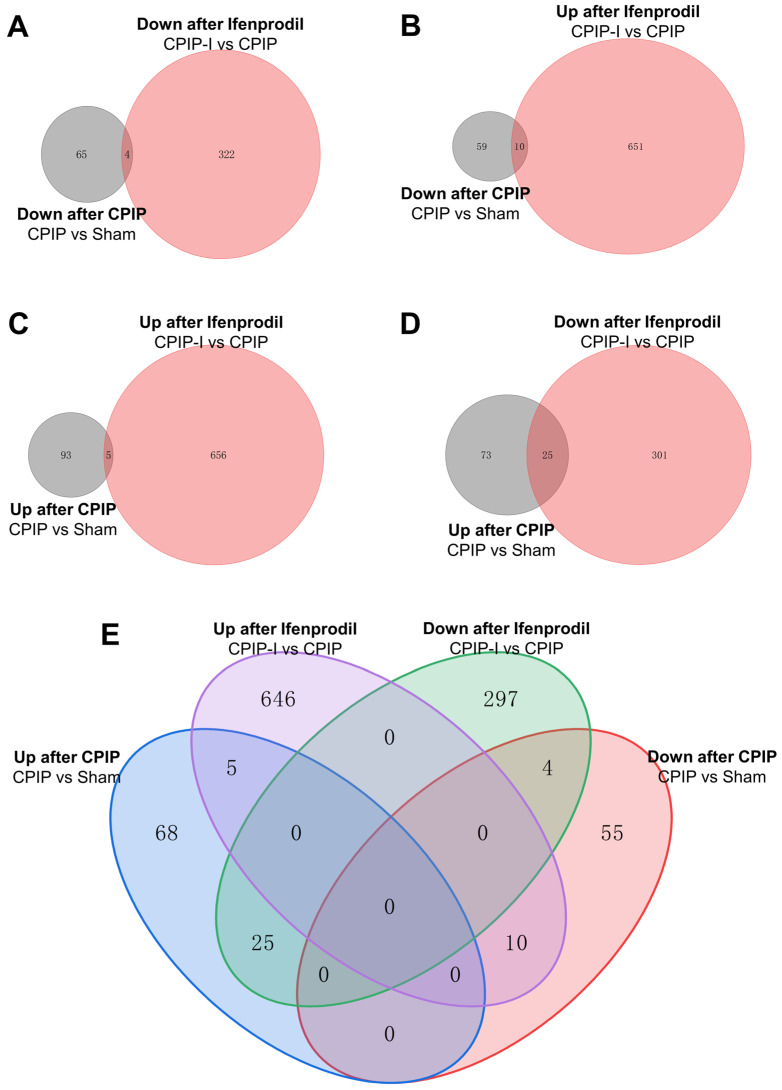
Overlapping of DEGs in DRGs of male rats from sham, CPIP, and CPIP-I groups. (**A**) A Venn diagram showing that 4 genes are downregulated in both CPIP and CPIP-I groups. (**B**) A Venn diagram showing that 10 genes are downregulated after CPIP and upregulated in CPIP-I groups. (**C**) A Venn diagram showing that 5 genes are upregulated in both CPIP and CPIP-I groups. (**D**) A Venn diagram showing that 25 genes are upregulated after CPIP and reversed in CPIP-I groups. (**E**) A Venn diagram showing the overlapping of DEGs among different groups. DEGs: differentially expressed genes; CPIP, chronic post-ischemia pain; I, ifenprodil.

Venn analysis was performed to elucidate the overlap of DEGs among different groups. The Venn diagrams presented in Figure 6 indicate that among the 69 downregulated DEGs in the CPIP group, 4 were further downregulated by intraplantar injection of ifenprodil (Figure 6A,E), whereas the downregulated expression of 10 was reversed (Figure 6B,E). Similarly, among the 98 upregulated DEGs in the CPIP group, the upregulated expression of 25 was reversed by intraplantar injection of ifenprodil (Figure 6D,E), and that of 5 was further upregulated (Figure 6C,E). Of the total set of 35 genes with reversed expression trends, 18 were encoded, the detailed information of which can be found in Supplementary Table S6.

### 3.5. Analysis of DEGs in the DRGs of CPIP Male Rats after Intraplantar Injection of Ifenprodil

**Figure 7:**
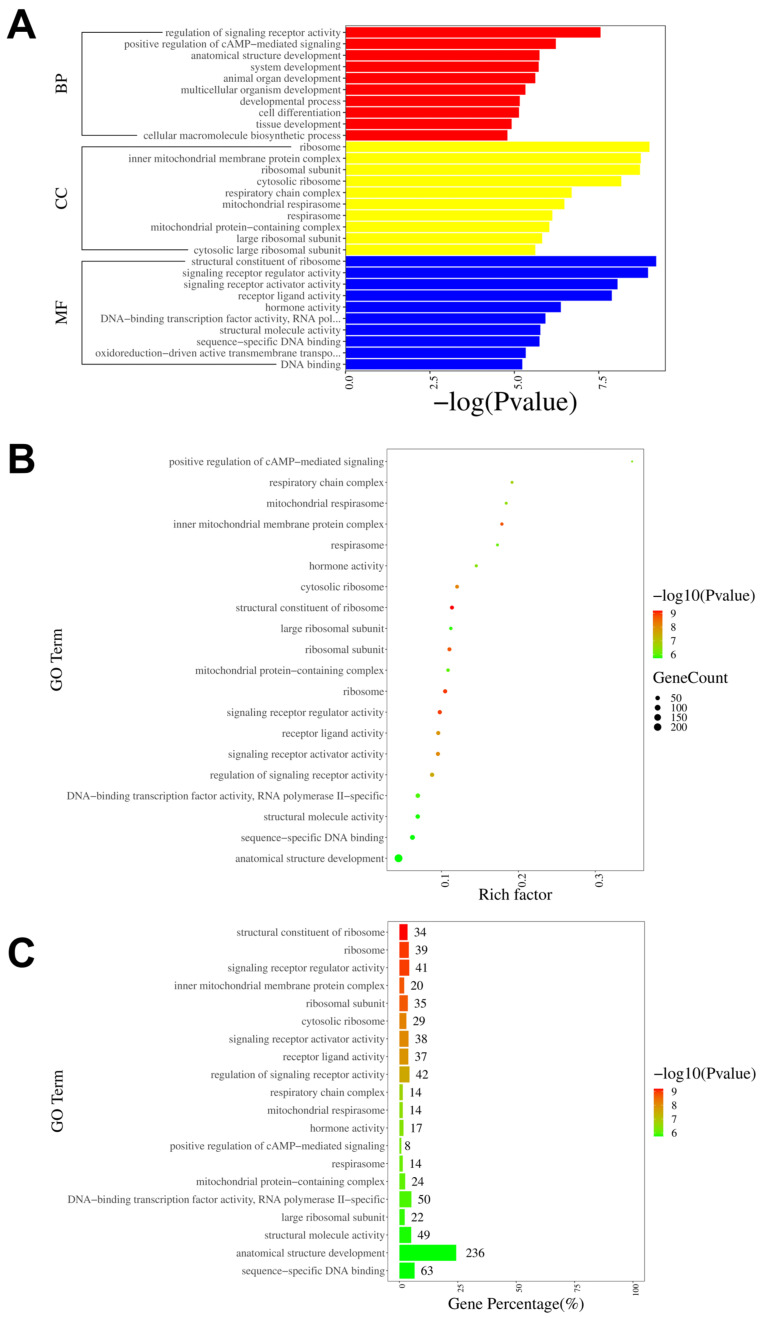
GO pathway analysis of DEGs in DRGs from male rats after intraplantar injection of ifenprodil. (**A**) A bar plot showing the top 10 significantly enriched pathways related to BP, CC, and MF in DEGs obtained from CPIP-I vs. CPIP. (**B**) A bubble plot showing the top 20 most significantly enriched GO pathways related to BP, CC, and MF in DEGs obtained from CPIP-I vs. CPIP. (**C**) A bar chart showing the top 20 pathways with the most enriched genes in DEGs obtained from CPIP-I vs. CPIP. DRG, dorsal root ganglion; GO: gene ontology; BP: biological process; CC: cellular components; MF: molecular function; DEGs: differentially expressed genes; CPIP, chronic post-ischemia pain; I, ifenprodil.

To further investigate the neural mechanisms associated with intraplantar ifenprodil injection, we conducted GO, KEGG, and GSEA analyses based on the genes showing differential expression between the CPIP-I and CPIP groups. The GO analysis revealed that the most significantly enriched biological processes, cellular components, and molecular functions of all DEGs were the regulation of signaling receptor activity, the structural constituent of ribosome, and ribosome, respectively (Figure 7A). The most enriched GO pathway was the positive regulation of cAMP-mediated signaling (Figure 7B), and the GO pathway with the most enriched genes was anatomical structure development (Figure 7C). The results of the GO analyses of the up- and downregulated DEGs are presented in Supplementary Figures S3 and S4, respectively.

**Figure 8:**
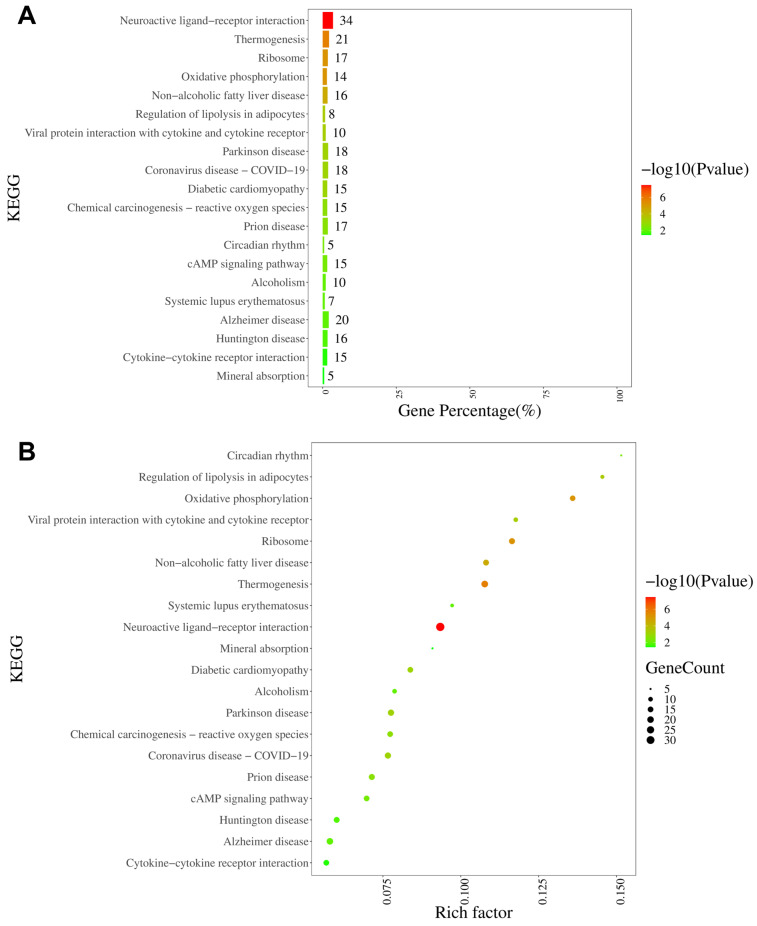
KEGG pathway analysis of DEGs in DRGs from male rats after intraplantar injection of ifenprodil. (**A**) A bar chart showing the top 20 pathways with the most enriched genes in DEGs obtained from CPIP-I vs. CPIP. (**B**) A bubble plot showing the top 20 most significantly enriched KEGG pathways in DEGs obtained from CPIP-I vs. CPIP. KEGG: Kyoto Encyclopedia of Genes and Genomes; DRG, dorsal root ganglion; DEGs: differentially expressed genes; CPIP, chronic post-ischemia pain; I, ifenprodil.

The KEGG analysis revealed that the neuroactive ligand–receptor interaction pathway was characterized by the largest number of enriched genes (34 genes), followed by thermogenesis, ribosomes, and oxidative phosphorylation (Figure 8A). Furthermore, on the basis of rich factor rankings, we identified the circadian rhythm pathway, regulation of lipolysis in adipocytes, and oxidative phosphorylation as the three most enriched KEGG pathways (Figure 8B). KEGG analyses of the up- and downregulated DEGs are shown in Supplementary Figures S5 and S6, respectively.

**Figure 9:**
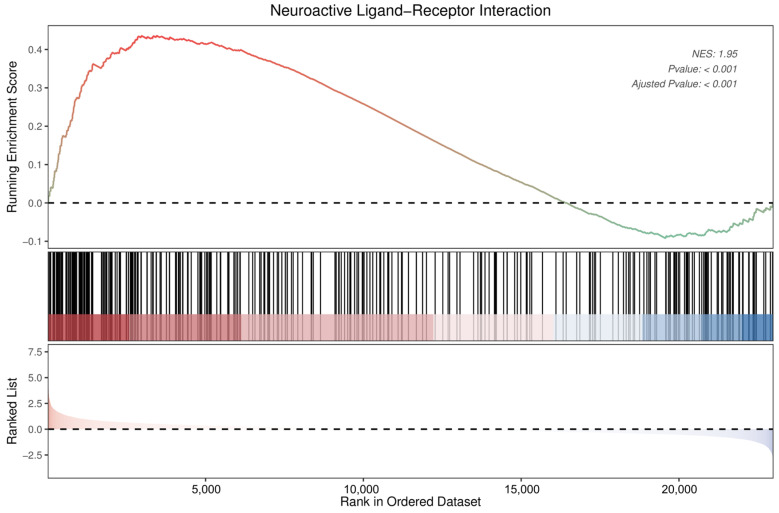
Enrichment plot for neuroactive ligand–receptor interactions of DEGs in DRGs of male rats after intraplantar injection of ifenprodil. DRG, dorsal root ganglion; DEGs: differentially expressed genes; NES: normalized enrichment score.

The GSEA also identified neuroactive ligand–receptor interactions as significantly enriched pathways (Figure 9). Detailed information on the 34 DEGs that were enriched in the neuroactive ligand–receptor interaction pathway, of which 5 were downregulated and 29 upregulated, is presented in Table 1. Among these, neuropeptide Y was found to show the most pronounced change (log2 fold change = 3.95), followed by relaxin/insulin-like family peptide receptor 2 (−2.65), adrenoceptor beta 3 (2.41), gonadotropin-releasing hormone 1 (−2.06), and somatostatin (2.02).

## 4. Discussion

An association between oxidative injury and chronic pain has previously been established, in which the outcomes of oxidative injury, such as the generation of ROS and lipid peroxidation, are assumed to contribute to the occurrence of chronic pain via the following mechanisms: direct activation of TRPA1 in peripheral nociceptive fibers [30], activation of glial cells in the spinal dorsal horn [31], modulation of synaptic plasticity in the spinal cord [32], and promotion of the production of allogenic substances [33]. For the purposes of the present study, we selected the production of ROS as an indicator of oxidative stress [34], 8-OHG was utilized as a marker of DNA/RNA oxidative damage [35], mitochondrial morphology and membrane potential were employed to assess mitochondrial function [36,37], and cell viability and apoptosis were measured to evaluate the overall level of cellular oxidative injury.

We established cell and rat models of CRPS and demonstrated that I/R caused oxidative injury to keratinocytes. CRPS reduced cell viability, induced mitochondrial damage, caused oxidative damage, and promoted apoptosis of keratinocytes both in vivo and in vitro. In contrast, CRPS promoted increases in the levels of intracellular ROS, and by regulating the expression of a series of antioxidant genes, it disrupted the balance between oxidation and antioxidation in keratinocytes. I/R is an important factor associated with CRPS that can activate and sensitize nociceptors [38]. Previous studies have demonstrated that I/R can disrupt the local oxidation–reduction process, trigger oxidative stress, and promote inflammatory progression [39]. Moreover, I/R can stimulate the overproduction of ROS and inflammatory factors, thereby contributing to hemodynamic disturbance and microvascular injury [40], which can in turn exacerbate ROS production and tissue hypoxia, consequently resulting in a vicious cycle of pain. Other studies have similarly revealed elevated levels of oxidation in the hind paws of rats with CPIP [16,41]. However, to the best of our knowledge, the present study is the first to focus on the oxidative injury of keratinocytes in the context of CRPS.

The Nrf2 signaling pathway is an essential component of the cellular defense system that orchestrates downstream signaling cascades that effectively counter oxidative stress [42,43]. Under normal conditions, Nrf2 is distributed in the cytoplasm, and its expression is maintained at stably low levels [44]. However, when cells are exposed to oxidative stress, Nrf2 is translocated to the nucleus, wherein it accumulates and activates the transcription of key antioxidant genes, including HO-1, SOD, and GSH [45]. To determine the antioxidant capacity of keratinocytes, we therefore assessed the expression of Nrf2 and its downstream genes, which revealed a significant reduction in the levels of Nrf2 in male rats with CPIP and in OGD/R HaCaT cells. Furthermore, we detected a downregulation in the expression of the downstream genes HO-1, SOD, and GSH in OGD/R keratinocytes, which was effectively reversed by antioxidant treatment. These findings thus provide evidence to indicate that the antioxidant capacity of keratinocytes is impaired in CRPS.

Glutamate receptors are widely expressed in the nervous system, participating in the regulation of synaptic plasticity and neuronal excitability. Depending on their structure and function, glutamate receptors can be classified into two major categories: ionotropic and metabotropic receptors. Metabotropic glutamate receptors (mGluRs) belong to the GPCR family C group and are composed of eight different subtypes (mGluR1-8). Ionotropic glutamate receptors include NMDA receptors, AMPA receptors, and KA receptors, which are ligand-gated ion channel receptors that regulate neuronal excitability and conductivity by controlling the entry of cations into the cell. Among them, NMDA receptors are complexes composed of NR1 and NR2 subunits, while the NR2 subunit includes four different isoforms (NR2A, NR2B, NR2C, and NR2D) [46]. NR2B is also associated with oxidative stress and neuronal loss in several diseases [47]. Antagonists of NR2B have been demonstrated to facilitate an upregulation of antioxidant proteins and elimination of lipid peroxidation products, thereby ameliorating oxidative damage in brain tissue and enhancing cognitive function in rats [18].

The next step in the present study was to investigate the involvement of NMDA receptors in keratinocyte oxidative injury in CRPS by administering both agonistic and antagonistic agents. Our findings indicate that ifenprodil mitigates hyperalgesia and oxidative injury in keratinocytes associated with CRPS, and that NMDA exacerbates these detrimental effects. Furthermore, the co-administration of NMDA and ifenprodil in the reoxygenation stage yields comparable results to OGD/R. Oxidative injury can initiate the activity of a cascade of transcription factors that facilitate the upregulation of pro-inflammatory mediators, thereby promoting the onset and progression of inflammation, which is closely associated with chronic pain [48,49,50,51].

Keratinocyte NR2B has been established to be is involved in the development of CRPS by promoting inflammation in the hind paw and DRG of male rats with CPIP. Consequently, it is plausible that the regulation of oxidative injury may serve as a mechanism whereby keratinocyte NMDA receptors play roles in CRPS [12].

To further elucidate the effects of intraplantar ifenprodil injection on male rats with CPIP, we conducted RNA-seq analysis of DRGs in different experimental groups. Our findings revealed that ifenprodil effectively reversed the altered gene expression that was observed in male rats with CPIP. Among these, guanylate cyclase [52,53], metallothionein [54,55,56], homeobox [57], olfactory receptor [58], prostaglandin-endoperoxide synthase 2 [59], matrix metallopeptidase 12 [60], and chrysrallin [61] play roles in different types of pain, including visceral, neuropathic, and cancer-related pain. These findings accordingly provide clues as to the possible mechanisms underlying the effects of peripheral ifenprodil treatment on CRPS.

On the basis of our KEGG analysis, we identified the neuroactive ligand–receptor interaction pathway in CPIP-I group rats as being enriched with a significantly higher number of genes than that in rats in the CPIP group. This pathway encompasses all receptor ligands that are located in the plasma membrane and that participate in both intracellular and extracellular signaling cascades. The aberrantly expressed genes included neuropeptide Y, relaxin/insulin-like family peptide receptor 2, adrenoceptor beta 3, gonadotropin-releasing hormone 1, somatostatin, adrenoceptor beta 1, cholecystokinin, and neuropeptide B. Enrichment of the same pathway has also been reported in studies that investigated different types of pain, including neuropathic pain, paclitaxel-induced peripheral neuropathy, and chronic compression injury pain [62,63,64].

However, despite our important findings in this study, the study does have certain limitations. Firstly, intraplantar administration of NMDA and ifenprodil may not only affect epidermal keratinocytes but also influence peripheral nerve fibers and other cells in the skin. To address this possibility, we conducted in vitro experiments using HaCaT cells to test our hypothesis. Additionally, we employed colocalized immunofluorescence with keratin to confirm the observed changes in keratinocytes during the in vivo studies. Secondly, our examination of the transcriptome profile of DRGs was essentially a preliminary assessment and requires further validation via PCR or protein-level analysis. Accordingly, our findings in this study provide initial insights into the mechanisms underlying CRPS within DRGs, and as such, further studies will be necessary to validate these findings and enable a more detailed characterization of the mechanisms involved. Furthermore, exclusively employing male rats in this study poses a substantial impact on our research, since extrapolating experimental findings solely from male subjects might not faithfully reflect the overall population owing to inherent variations. Consequently, it is imperative that forthcoming studies utilizing the CPIP incorporate both male and female models.

## 5. Conclusions

**Figure 10:**
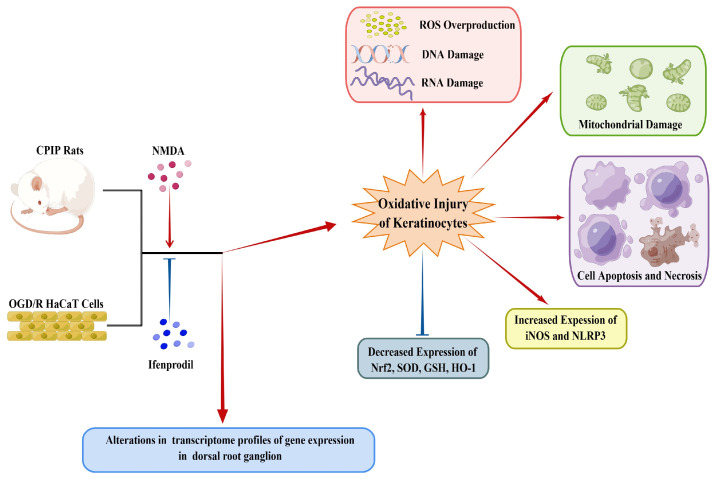
A schematic model showing the occurrence of oxidative injury in keratinocytes during CRPS, which can be regulated by NMDA receptors. CRPS, complex regional pain syndrome; CPIP, chronic post-ischemia pain; OGD/R, oxygen–glucose deprivation/reoxygenation; NLRP3, NOD-like receptor thermal protein domain associated protein 3; Nrf-2, nuclear factor erythroid 2-related factor 2; iNOS, inducible nitric oxide synthase; HO-1, heme oxygenase-1; GSH, glutathione; SOD, superoxide dismutase; ROS, reactive oxygen species (by Figdraw, https://www.figdraw.com).

As depicted in Figure 10, this study demonstrates the occurrence of oxidative injury in keratinocytes of CRPS and highlights the regulatory roles played by NMDA receptors in this process. Local application of ifenprodil was found to influence neural function and gene expression in the DRG of male rats. Further investigations are required to elucidate the interactions between keratinocytes and sensory neurons in CRPS.

## Figures and Tables

**Table 1:** Detailed information for DEGs in neuroactive ligand–receptor interactions.

Gene_ID	Gene Symbol	Full Gene Name	Log_2_ Fold Change (CPIP-I/CPIP)	*p* Value
ENSRNOG00000047545	Adra2a	adrenoceptor alpha 2A	1.05	2.40 × 10^−7^
ENSRNOG00000017002	Adrb1	adrenoceptor beta 1	1.89	5.76 × 10^−6^
ENSRNOG00000012674	Adrb3	adrenoceptor beta 3	2.41	4.00 × 10^−3^
ENSRNOG00000011074	Calcb	calcitonin-related polypeptide, beta	1.02	1.13 × 10^−21^
ENSRNOG00000019321	Cck	cholecystokinin	1.63	1.04 × 10^−4^
ENSRNOG00000017556	Chrm4	cholinergic receptor, muscarinic 4	1.14	3.32 × 10^−6^
ENSRNOG00000006397	Chrm5	cholinergic receptor, muscarinic 5	−1.17	2.03 × 10^−2^
ENSRNOG00000012703	Crh	corticotropin-releasing hormone	1.28	1.45 × 10^−2^
ENSRNOG00000011145	Crhr2	corticotropin-releasing hormone receptor 2	1.16	1.84 × 10^−4^
ENSRNOG00000008428	Drd2	dopamine receptor D2	1.13	4.47 × 10^−3^
ENSRNOG00000013433	Gnrh1	gonadotropin-releasing hormone 1	−2.06	1.91 × 10^−2^
ENSRNOG00000024030	Gpr35	G protein-coupled receptor 35	1.24	6.08 × 10^−7^
ENSRNOG00000010254	Htr1a	5-hydroxytryptamine receptor 1A	1.32	1.88 × 10^−4^
ENSRNOG00000002549	Htr5b	5-hydroxytryptamine (serotonin) receptor 5B	1.02	2.95 × 10^−4^
ENSRNOG00000047040	Lhb	luteinizing hormone beta polypeptide	1.20	1.93 × 10^−2^
ENSRNOG00000015260	Lpar3	lysophosphatidic acid receptor 3	1.13	2.91 × 10^−3^
ENSRNOG00000036685	Npb	neuropeptide B	1.91	2.38 × 10^−6^
ENSRNOG00000012390	Npw	neuropeptide W	1.59	7.06 × 10^−5^
ENSRNOG00000009768	Npy	neuropeptide Y	3.95	5.88 × 10^−25^
ENSRNOG00000004179	Nts	neurotensin	−1.18	3.36 × 10^−3^
ENSRNOG00000037839	P2ry10	purinergic receptor P2Y10	−1.19	3.31 × 10^−2^
ENSRNOG00000014231	Pnoc	prepronociceptin	1.04	3.74 × 10^−3^
ENSRNOG00000009922	Prlhr	prolactin releasing hormone receptor	1.76	1.94 × 10^−2^
ENSRNOG00000031535	Ptgdr	prostaglandin D2 receptor	1.34	3.09 × 10^−3^
ENSRNOG00000031307	Ptgdrl	prostaglandin D2 receptor-like	1.47	3.93 × 10^−8^
ENSRNOG00000000897	Rxfp2	relaxin/insulin-like family peptide receptor 2	−2.65	8.32× 10^−3^
ENSRNOG00000023126	Rxfp3	relaxin/insulin-like family peptide receptor 3	1.95	2.04 × 10^−2^
ENSRNOG00000005370	S1pr4	sphingosine-1-phosphate receptor 4	1.16	4.59 × 10^−2^
ENSRNOG00000020901	S1pr5	sphingosine-1-phosphate receptor 5	1.57	8.75 × 10^−3^
ENSRNOG00000001837	Sst	somatostatin	2.02	2.56 × 10^−6^
ENSRNOG00000004641	Sstr4	somatostatin receptor 4	1.09	4.59 × 10^−3^
ENSRNOG00000003972	Tshr	thyroid-stimulating hormone receptor	1.50	3.23 × 10^−2^
ENSRNOG00000006090	Ucn	urocortin	1.99	3.64 × 10^−2^
ENSRNOG00000001416	Vgf	VGF nerve growth factor inducible	1.18	6.99 × 10^−7^

## Data Availability

The datasets used and/or analyzed in the current study are available from the corresponding author upon reasonable request.

## Supplementary Materials

The following supporting information can be downloaded at https://www.mdpi.com/article/10.3390/antiox13020244/s1: Supplementary Figure S1: Timeline of animal experiments; Supplementary Figure S2. ROS levels in HaCaT cells after different treatments; Supplementary Figure S3: GO pathway analysis of upregulated DEGs in DRGs from male rats after intraplantar injection of ifenprodil; Supplementary Figure S4: GO pathway analysis of downregulated DEGs in DRGs from male rats after intraplantar injection of ifenprodil; Supplementary Figure S5: KEGG pathway analysis of upregulated DEGs in DRGs from male rats after intraplantar injection of ifenprodil; Supplementary Figure S6: KEGG pathway analysis of downregulated DEGs in DRGs from male rats after intraplantar injection of ifenprodil; Supplementary Table S1: Antibodies used in immunofluorescence; Supplementary Table S2: List of the primer sequences for RT-qPCR; Supplementary Table S3: Information on total reads and mapping ratio for sham, CPIP, and CPIP+I in RNA-Seq; Supplementary Table S4: Detailed information about the top 20 upregulated DEGs; Supplementary Table S5: Detailed information about the top 20 downregulated DEGs; Supplementary Table S6: Detailed information on reversed encoding genes in the CPIP+I group.

## References

[1] Nicholas M., Vlaeyen J.W.S., Rief W., Barke A., Aziz Q., Benoliel R., Cohen M., Evers S., Giamberardino M.A., Goebel A. (2019). The IASP classification of chronic pain for ICD-11: Chronic primary pain. Pain.

[2] David Clark J., Tawfik V.L., Tajerian M., Kingery W.S. (2018). Autoinflammatory and autoimmune contributions to complex regional pain syndrome. Mol. Pain.

[3] Birklein F., Ajit S.K., Goebel A., Perez R., Sommer C. (2018). Complex regional pain syndrome—Phenotypic characteristics and potential biomarkers. Nat. Rev. Neurol..

[4] Arcidiacono U.A., Armocida D., Pesce A., Maiotti M., Proietti L., D’Andrea G., Santoro A., Frati A. (2022). Complex Regional Pain Syndrome after Spine Surgery: A Rare Complication in Mini-Invasive Lumbar Spine Surgery: An Updated Comprehensive Review. J. Clin. Med..

[5] Kessler A., Yoo M., Calisoff R. (2020). Complex regional pain syndrome: An updated comprehensive review. NeuroRehabilitation.

[6] Shim H., Rose J., Halle S., Shekane P. (2019). Complex regional pain syndrome: A narrative review for the practising clinician. Br. J. Anaesth..

[7] Baronio M., Sadia H., Paolacci S., Prestamburgo D., Miotti D., Guardamagna V.A., Natalini G., Sullivan S.G.B., Bertelli M. (2020). Molecular Aspects of Regional Pain Syndrome. Pain Res. Manag..

[8] Zhu H., Wen B., Xu L., Huang Y. (2023). Identification of Potential Inflammation-Related Genes and Key Pathways Associated with Complex Regional Pain Syndrome. Biomolecules.

[9] Wen B., Pan Y., Cheng J., Xu L., Xu J. (2023). The Role of Neuroinflammation in Complex Regional Pain Syndrome: A Comprehensive Review. J. Pain Res..

[10] Baron R., Hans G., Dickenson A.H. (2013). Peripheral input and its importance for central sensitization. Ann. Neurol..

[11] Kuner R. (2010). Central mechanisms of pathological pain. Nat. Med..

[12] Xu X., Yu C., Xu L., Xu J. (2022). Emerging roles of keratinocytes in nociceptive transduction and regulation. Front. Mol. Neurosci..

[13] Xu X., Tao X., Huang P., Lin F., Liu Q., Xu L., Xu J., Huang Y. (2020). N-methyl-d-aspartate receptor subunit 2B on keratinocyte mediates peripheral and central sensitization in chronic post-ischemic pain in male rats. Brain Behav. Immun..

[14] Kaushik A.S., Strath L.J., Sorge R.E. (2020). Dietary Interventions for Treatment of Chronic Pain: Oxidative Stress and Inflammation. Pain Ther..

[15] Hsiao H.T., Lin Y.C., Wang J.C., Tsai Y.C., Liu Y.C. (2016). Hypoxia inducible factor-1α inhibition produced anti-allodynia effect and suppressed inflammatory cytokine production in early stage of mouse complex regional pain syndrome model. Clin. Exp. Pharmacol. Physiol..

[16] Li X., Yin C., Hu Q., Wang J., Nie H., Liu B., Tai Y., Fang J., Du J., Shao X. (2022). Nrf2 Activation Mediates Antiallodynic Effect of Electroacupuncture on a Rat Model of Complex Regional Pain Syndrome Type-I through Reducing Local Oxidative Stress and Inflammation. Oxid. Med. Cell. Longev..

[17] Coderre T.J., Xanthos D.N., Francis L., Bennett G.J. (2004). Chronic post-ischemia pain (CPIP): A novel animal model of complex regional pain syndrome-type I (CRPS-I; reflex sympathetic dystrophy) produced by prolonged hindpaw ischemia and reperfusion in the rat. Pain.

[18] Gao X., Chen F., Xu X., Liu J., Dong F., Liu Y. (2022). Ro25-6981 alleviates neuronal damage and improves cognitive deficits by attenuating oxidative stress via the Nrf2/ARE pathway in ischemia/reperfusion rats. J. Stroke Cerebrovasc. Dis..

[19] Fischer G., Mutel V., Trube G., Malherbe P., Kew J.N., Mohacsi E., Heitz M.P., Kemp J.A. (1997). Ro 25-6981, a highly potent and selective blocker of N-methyl-D-aspartate receptors containing the NR2B subunit. Characterization in vitro. J. Pharmacol. Exp. Ther..

[20] Yang S., Park S.H., Oh S.W., Kwon K., Yu E., Lee C.W., Son Y.K., Kim C., Lee B.H., Cho J.Y. (2022). Antioxidant Activities and Mechanisms of Tomentosin in Human Keratinocytes. Antioxidants.

[21] Jiang B.W., Zhang W.J., Wang Y., Tan L.P., Bao Y.L., Song Z.B., Yu C.L., Wang S.Y., Liu L., Li Y.X. (2020). Convallatoxin induces HaCaT cell necroptosis and ameliorates skin lesions in psoriasis-like mouse models. Biomed. Pharmacother..

[22] Lee S.Y., Kim C.H., Hwang B.S., Choi K.M., Yang I.J., Kim G.Y., Choi Y.H., Park C., Jeong J.W. (2020). Protective Effects of Oenothera biennis against Hydrogen Peroxide-Induced Oxidative Stress and Cell Death in Skin Keratinocytes. Life.

[23] Kim J.S., Lim S.S. (2022). LED Light-Induced ROS Differentially Regulates Focal Adhesion Kinase Activity in HaCaT Cell Viability. Curr. Issues Mol. Biol..

[24] Bruehl S., Warner D.S. (2010). An Update on the Pathophysiology of Complex Regional Pain Syndrome. Anesthesiology.

[25] Ragavendran J.V., Laferrière A., Khorashadi M., Coderre T.J. (2014). Pentoxifylline reduces chronic post-ischaemia pain by alleviating microvascular dysfunction. Eur. J. Pain.

[26] Tang Y., Liu L., Xu D., Zhang W., Zhang Y., Zhou J., Huang W. (2018). Interaction between astrocytic colony stimulating factor and its receptor on microglia mediates central sensitization and behavioral hypersensitivity in chronic post ischemic pain model. Brain Behav. Immun..

[27] Liu Y., Liang Y., Gao M., Li Y., Zhao T., Zhao Y. (2021). Animal Models of Complex Regional Pain Syndrome Type I. J. Pain Res..

[28] Arifin W.N., Zahiruddin W.M. (2017). Sample Size Calculation in Animal Studies Using Resource Equation Approach. Malays. J. Med. Sci..

[29] Sadler K.E., Patitucci T.N., Stucky C.L., Seal R.P. (2022). Ex Vivo Skin-Teased Fiber Recordings from Tibial NerveTibial nerves. Contemporary Approaches to the Study of Pain: From Molecules to Neural Networks.

[30] Liu B., Tai Y., Caceres A.I., Achanta S., Balakrishna S., Shao X., Fang J., Jordt S.E. (2016). Oxidized Phospholipid OxPAPC Activates TRPA1 and Contributes to Chronic Inflammatory Pain in Mice. PLoS ONE.

[31] Shi Y., Yuan S., Tang S.J. (2021). Reactive Oxygen Species (ROS) are Critical for Morphine Exacerbation of HIV-1 gp120-Induced Pain. J. Neuroimmune Pharmacol..

[32] Bittar A., Jun J., La J.H., Wang J., Leem J.W., Chung J.M. (2017). Reactive oxygen species affect spinal cell type-specific synaptic plasticity in a model of neuropathic pain. Pain.

[33] Zheng J., Zhang J., Zhang X., Guo Z., Wu W., Chen Z., Li J. (2021). Reactive Oxygen Species Mediate Low Back Pain by Upregulating Substance P in Intervertebral Disc Degeneration. Oxid. Med. Cell. Longev..

[34] Zhao Y., Xu J. (2020). Sanggenon C Ameliorates Cerebral Ischemia-Reperfusion Injury by Inhibiting Inflammation and Oxidative Stress through Regulating RhoA-ROCK Signaling. Inflammation.

[35] Li S., Jiang D., Rosenkrans Z.T., Barnhart T.E., Ehlerding E.B., Ni D., Engle J.W., Cai W. (2019). Aptamer-Conjugated Framework Nucleic Acids for the Repair of Cerebral Ischemia-Reperfusion Injury. Nano Lett..

[36] Mukherjee A., Sarkar S., Jana S., Swarnakar S., Das N. (2019). Neuro-protective role of nanocapsulated curcumin against cerebral ischemia-reperfusion induced oxidative injury. Brain Res..

[37] He R., Jiang Y., Shi Y., Liang J., Zhao L. (2020). Curcumin-laden exosomes target ischemic brain tissue and alleviate cerebral ischemia-reperfusion injury by inhibiting ROS-mediated mitochondrial apoptosis. Mater. Sci. Eng. C Mater. Biol. Appl..

[38] Groeneweg G., Huygen F.J., Coderre T.J., Zijlstra F.J. (2009). Regulation of peripheral blood flow in complex regional pain syndrome: Clinical implication for symptomatic relief and pain management. BMC Musculoskelet. Disord..

[39] Yang J., Qi J., Xiu B., Yang B., Niu C., Yang H. (2019). Reactive Oxygen Species Play a Biphasic Role in Brain Ischemia. J. Investig. Surg..

[40] Coderre T.J., Bennett G.J. (2010). A hypothesis for the cause of complex regional pain syndrome-type I (reflex sympathetic dystrophy): Pain due to deep-tissue microvascular pathology. Pain Med..

[41] Klafke J.Z., da Silva M.A., Rossato M.F., de Prá S.D., Rigo F.K., Walker C.I., Bochi G.V., Moresco R.N., Ferreira J., Trevisan G. (2016). Acute and chronic nociceptive phases observed in a rat hind paw ischemia/reperfusion model depend on different mechanisms. Pflugers Arch..

[42] Motterlini R., Foresti R. (2014). Heme oxygenase-1 as a target for drug discovery. Antioxid. Redox Signal..

[43] Kesherwani V., Atif F., Yousuf S., Agrawal S.K. (2013). Resveratrol protects spinal cord dorsal column from hypoxic injury by activating Nrf-2. Neuroscience.

[44] Shaw P., Chattopadhyay A. (2020). Nrf2-ARE signaling in cellular protection: Mechanism of action and the regulatory mechanisms. J. Cell. Physiol..

[45] Bellezza I., Giambanco I., Minelli A., Donato R. (2018). Nrf2-Keap1 signaling in oxidative and reductive stress. Biochim. Biophys. Acta Mol. Cell Res..

[46] Paoletti P., Bellone C., Zhou Q. (2013). NMDA receptor subunit diversity: Impact on receptor properties, synaptic plasticity and disease. Nat. Rev. Neurosci..

[47] Zhu X., Dong J., Shen K., Bai Y., Zhang Y., Lv X., Chao J., Yao H. (2015). NMDA receptor NR2B subunits contribute to PTZ-kindling-induced hippocampal astrocytosis and oxidative stress. Brain Res. Bull..

[48] Uçeyler N., Eberle T., Rolke R., Birklein F., Sommer C. (2007). Differential expression patterns of cytokines in complex regional pain syndrome. Pain.

[49] Sandireddy R., Yerra V.G., Areti A., Komirishetty P., Kumar A. (2014). Neuroinflammation and oxidative stress in diabetic neuropathy: Futuristic strategies based on these targets. Int. J. Endocrinol..

[50] Sibille K.T., Steingrímsdóttir Ó.A., Fillingim R.B., Stubhaug A., Schirmer H., Chen H., McEwen B.S., Nielsen C.S. (2016). Investigating the Burden of Chronic Pain: An Inflammatory and Metabolic Composite. Pain Res. Manag..

[51] Allison D.J., Thomas A., Beaudry K., Ditor D.S. (2016). Targeting inflammation as a treatment modality for neuropathic pain in spinal cord injury: A randomized clinical trial. J. Neuroinflamm..

[52] Hannig G., Tchernychev B., Kurtz C.B., Bryant A.P., Currie M.G., Silos-Santiago I. (2014). Guanylate cyclase-C/cGMP: An emerging pathway in the regulation of visceral pain. Front. Mol. Neurosci..

[53] Silos-Santiago I., Hannig G., Eutamene H., Ustinova E.E., Bernier S.G., Ge P., Graul C., Jacobson S., Jin H., Liong E. (2013). Gastrointestinal pain: Unraveling a novel endogenous pathway through uroguanylin/guanylate cyclase-C/cGMP activation. Pain.

[54] Huang X., Deng J., Xu T., Xin W., Zhang Y., Ruan X. (2021). Downregulation of metallothionein-2 contributes to oxaliplatin-induced neuropathic pain. J. Neuroinflamm..

[55] Oki G., Wada T., Iba K., Aiki H., Sasaki K., Imai S.I., Sohma H., Matsumoto K., Yamaguchi M., Fujimiya M. (2012). Metallothionein deficiency in the injured peripheral nerves of complex regional pain syndrome as revealed by proteomics. Pain.

[56] Heo J.H., Lee S.H., Chang K.H., Han E.H., Lee S.G., Choi D.W., Kim S.W. (2013). Identification of differentially expressed genes by gabapentin in cultured dorsal root ganglion in a rat neuropathic pain model. Biomol. Ther..

[57] Zhang J., Mei Z., Yao W., Zhao C., Wu S., Ouyang J. (2023). SIX1 induced HULC modulates neuropathic pain and Schwann cell oxidative stress after sciatic nerve injury. Gene.

[58] Lee E., Takita C., Wright J.L., Slifer S.H., Martin E.R., Urbanic J.J., Langefeld C.D., Lesser G.J., Shaw E.G., Hu J.J. (2019). Genome-wide enriched pathway analysis of acute post-radiotherapy pain in breast cancer patients: A prospective cohort study. Hum. Genom..

[59] Chen J., Guo P., Liu X., Liao H., Chen K., Wang Y., Qin J., Yang F. (2023). Sinomenine alleviates diabetic peripheral neuropathic pain through inhibition of the inositol-requiring enzyme 1 alpha-X-box binding protein 1 pathway by downregulating prostaglandin-endoperoxide synthase 2. J. Diabetes Investig..

[60] Husain S.F., Lam R.W.M., Hu T., Ng M.W.F., Liau Z.Q.G., Nagata K., Khanna S., Lam Y., Bhakoo K., Ho R.C.M. (2019). Locating the Site of Neuropathic Pain In Vivo Using MMP-12-Targeted Magnetic Nanoparticles. Pain Res. Manag..

[61] Lim E.F., Hoghooghi V., Hagen K.M., Kapoor K., Frederick A., Finlay T.M., Ousman S.S. (2021). Presence and activation of pro-inflammatory macrophages are associated with CRYAB expression in vitro and after peripheral nerve injury. J. Neuroinflamm..

[62] Cui C.Y., Liu X., Peng M.H., Liu Q., Zhang Y. (2022). Identification of key candidate genes and biological pathways in neuropathic pain. Comput. Biol. Med..

[63] Sun W., Yang S., Wu S., Ba X., Xiong D., Xiao L., Hao Y. (2023). Transcriptome analysis reveals dysregulation of inflammatory and neuronal function in dorsal root ganglion of paclitaxel-induced peripheral neuropathy rats. Mol. Pain.

[64] Du Z., Yin S., Song X., Zhang L., Yue S., Jia X., Zhang Y. (2020). Identification of Differentially Expressed Genes and Key Pathways in the Dorsal Root Ganglion After Chronic Compression. Front. Mol. Neurosci..

