# Supplementary material for: NMDA Receptors Regulate Oxidative Damage in Keratinocytes during Complex Regional Pain Syndrome in HaCaT Cells and Male Rats
Source: Antioxidants (Basel). 2024 Feb 18;13(2):244. doi: 10.3390/antiox13020244 (PMC10886417; doi:10.3390/antiox13020244)
Supplement: Supplementary file 1 [file antioxidants-13-00244-s001.zip › 2.Supplementary Tables.pdf]

Supplementary Table S1. Antibodies used in immunofluorescence.

Antibody	Function	Host	Company	Catalog number	Dilution
8-OHG	a marker of DNA/RNA damage caused by oxidation	Mouse	Abcam	Ab62623	1:200
NLRP3	an inflammasome that facilitates the secretion of proinflammatory cytokines	Rabbit	Invitrogen	SC-06-23	1:100
Nrf-2	a transcription factor that stimulates the transcription of key antioxidant genes	Rabbit	Proteintech	16396-1-AP	1:100
iNOS	inducible nitric oxide synthase that upregulated by pathological conditions and promote the production of	Rabbit	Proteintech	18985-1-AP	1:100
Cytokeratin 14	a marker of keratinocytes	Rabbit	Proteintech	10143-1-AP	1:200
pan-keratin	a marker of keratinocytes	Mouse	Abcam	ab8068	1:50
488-conjugated Anti-Mouse IgG	secondary immunofluorescence antibody	Goat	Proteintech	SA00013-1	1:500
594-conjugated Anti-Rabbit IgG	secondary immunofluorescence antibody	Goat	Proteintech	SA00013-4	1:500

Abbreviations: 8-OHG, 8-hydroxy-2 deoxyguanosine; NLRP3, NOD-like receptor thermal protein domain associated protein 3; Nrf-2, Nuclear factor erythroid 2-related factor 2; iNOS, inducible nitric oxide synthase.

Supplementary Table S2. List of the primer sequence for RT-qPCR.

Target	Species	Function	Direction	Primer sequence 5′-3′	Product length(bp)
Nrf2	Human	a transcription factor that stimulates the transcription of key antioxidant genes	Forward	GTTCCAAGTCCAGAAGCCAAACTG	143
			Reverse	GTGGAGAGGATGCTGCTGAAGG	
HO-1	Human	hemeoxygenase-1, an antioxidant gene that has a cytoprotective effect	Forward	ACTGCGTTCCTGCTCAACATCC	76
			Reverse	ACTGCGTTCCTGCTCAACATCC	
GSH	Human	glutathione, combines with free radicals to exert a powerful antioxidant effect	Forward	CGAGAATGTGGCGTCCCTCTG	144
			Reverse	TCGTTCTTGGCGTTCTCCTGATG	
SOD	Human	superoxide dismutase, a crucial enzyme with antioxidative properties.	Forward	TCGTTCTTGGCGTTCTCCTGATG	75
			Reverse	TCGTTCTTGGCGTTCTCCTGATG	
GAPDH	Human	widely distributed in various tissues and used as a standardized internal reference	Forward	GCACCGTCAAGGCTGAGAAC	138
			Reverse	TGGTGAAGACGCCAGTGGA	

Abbreviations: Nrf2, Nuclear factor erythroid 2-related factor 2; HO-1, Heme Oxygenase-1; GSH, Glutathione; SOD, Superoxide Dismutase; GAPDH, glyceraldehyde-3-phosphate dehydrogenase.

Supplementary Table S3 The information on total reads and mapping ratio for Sham, CPIP, and CPIP+I in RNA-Seq

Sample	Total raw reads	Total clean reads	Average length	Clean reads Q30 (%)	Clean reads ratio (%)	Mapped ratio (%)
Sham 1	54,665,842	52,350,888	149.2	94.9	95.8	96.2
Sham 2	65,472,702	63,650,948	149.1	94.0	97.2	95.7
Sham 3	68,785,886	67,076,022	149.1	94.2	97.5	95.5
CPIP 1	75,242,610	70,530,908	149.0	94.9	93.7	96.9
CPIP 2	81,256,734	78,292,910	149.2	94.8	96.4	97.2
CPIP 3	75,964,080	73,824,004	149.2	94.3	97.2	95.7
CPIP+I 1	51,505,388	50,742,640	149.3	92.5	98.5	95.6
CPIP+I 2	59,183,222	58,276,128	149.5	92.9	98.5	96.0
CPIP+I 3	61,102,598	60,151,196	149.5	92.9	98.4	96.1

CPIP, Chronic postischemia pain; I, ifenprodil; RNA-Seq, RNA-sequencing.

Supplementary Table S4. Detailed information about the top 20 upregulated DEGs

	Gene ID	Gene symbol	Full gene name	Log_2 fold change (CPIP-I/CPIP)	P value
1	ENSRNOG00000004757	Tmem158	transmembrane protein 158	4.57	2.87E-247
2	ENSRNOG00000005158	Slc24a5	solute carrier family 24 member 5	4.28	2.30E-07
3	ENSRNOG00000057335	Clec1b	C-type lectin domain family 1, member B	4.06	5.90E-07
4	ENSRNOG00000036864	Actl10	actin-like 10	3.94	1.91E-06
5	ENSRNOG00000046449	Npy	neuropeptide Y	3.79	8.53E-23
6	ENSRNOG00000009326	Smcp	sperm mitochondria-associated cysteine-rich protein	3.68	8.01E-06
7	ENSRNOG00000048651	Nrtn	neurturin	3.50	1.35E-14
8	ENSRNOG00000055936	Trnp1	TMF1-regulated nuclear protein 1	3.47	1.22E-204
9	ENSRNOG00000021016	Ntn5	netrin 5	3.45	1.60E-04
10	ENSRNOG00000057315	Kcnh3	potassium voltage-gated channel subfamily H member 3	3.35	1.71E-04
11	ENSRNOG00000004719	Pp2d1	protein phosphatase 2C-like domain containing 1	3.25	1.14E-04
12	ENSRNOG00000008849	Guca2a	guanylate cyclase activator 2A	3.21	1.17E-04
13	ENSRNOG00000031955	Calml3	calmodulin-like 3	3.11	1.41E-03
14	ENSRNOG00000026387	Ms4a12	membrane spanning 4-domains A12	2.96	5.29E-04
15	ENSRNOG00000034236	C2cd4a	C2 calcium-dependent domain containing 4A	2.95	5.25E-10
16	ENSRNOG00000010972	Neurog2	neurogenin 2	2.90	6.81E-04
17	ENSRNOG00000003109	Btbd17	BTB domain containing 17	2.89	7.58E-05
18	ENSRNOG00000048875	Znrf4	zinc and ring finger 4	2.87	7.83E-04
19	ENSRNOG00000008518	Gsg1	germ cell associated 1	2.86	2.07E-03
20	ENSRNOG00000054250	Fam186b	family with sequence similarity 186, member B	2.78	2.59E-04

Supplementary Table S5. Detailed information about the top 20 downregulated DEGs

	Gene ID	Gene symbol	Full gene name	Log2 fold change CPIP-I/CPIP	P value
1	ENSRNOG00000012566	Kcnv2	potassium voltage-gated channel modifier subfamily V member 2	-4.02	2.55E-02
2	ENSRNOG00000015972	Ano5	anoctamin 5	-3.54	4.61E-05
3	ENSRNOG00000025200	LOC102553785	uncharacterized LOC102553785	-3.03	4.11E-04
4	ENSRNOG00000032777	Rpl26-ps1	ribosomal protein L26, pseudogene 1	-2.93	8.36E-04
5	ENSRNOG00000003790	Mael	maelstrom spermatogenic transposon silencer	-2.74	8.47E-03
6	ENSRNOG00000042533	Accsl	1-aminocyclopropane-1-carboxylate synthase-like	-2.66	8.66E-03
7	ENSRNOG00000000897	Rxfp2	relaxin/insulin-like family peptide receptor 2	-2.65	8.32E-03
8	ENSRNOG00000028721	Otor	otoraplin	-2.59	1.22E-02
9	ENSRNOG00000021160	Hormad1	HORMA domain containing 1	-2.57	1.60E-03
10	ENSRNOG00000057989	Zp2	zona pellucida glycoprotein 2	-2.57	9.60E-04
11	ENSRNOG00000005178	Cstdc2	cystatin domain containing 2	-2.49	9.59E-03
12	ENSRNOG00000026315	Taf7l	TATA-box binding protein associated factor 7-like	-2.42	1.07E-02
13	ENSRNOG00000001942	Smr3b	submaxillary gland androgen regulated protein 3B	-2.41	2.06E-02
14	ENSRNOG00000011823	Tfap2b	transcription factor AP-2 beta	-2.39	4.17E-03
15	ENSRNOG00000001360	Stag3	stromal antigen 3	-2.39	3.88E-03
16	ENSRNOG00000029591	Pkdrej	polycystin (PKD) family receptor for egg jelly	-2.37	6.14E-03
17	ENSRNOG00000022937	Bpifc	BPI fold containing family C	-2.37	1.10E-02
18	ENSRNOG00000030431	RT1-Db2	RT1 class II, locus Db2	-2.35	8.34E-03
19	ENSRNOG00000002754	Areg	amphiregulin	-2.34	1.24E-02
20	ENSRNOG00000059551	LOC103689976	sperm motility kinase 3-like	-2.32	1.46E-02

Supplementary Table S6. The detailed information of reversed encoding genes in the CPIP+I group.

Gene_ID	Gene symbol	Full gene name	Log2 fold change (CPIP/Sham)	Log2 fold change (CPIP-I/CPIP)	Reverse rate
ENSRNOG00000011823	Tfap2b	transcription factor AP-2 beta	1.16	-2.39	2.06
ENSRNOG00000017248	Prkag3	protein kinase AMP-activated non-catalytic subunit gamma 3	1.31	-1.99	1.52
ENSRNOG00000008849	Guca2a	guanylate cyclase activator 2A	-2.39	3.21	1.35
ENSRNOG00000038047	Mt1-ps3	metallothionein 1, pseudogene 3	-1.32	1.75	1.33
ENSRNOG00000059870	Hoxa11	homeobox A11	1.50	-1.92	1.28
ENSRNOG00000021332	Olr86	olfactory receptor 86	-1.59	1.85	1.16
ENSRNOG00000002525	Ptgs2	prostaglandin-endoperoxide synthase 2	1.10	-1.25	1.14
ENSRNOG00000005178	Cstdc2	cystatin domain containing 2	2.28	-2.39	1.05
ENSRNOG00000030187	Mmp12	matrix metallopeptidase 12	2.05	-2.02	0.99
ENSRNOG00000047673	Crybb3	crystallin, beta B3	1.81	-1.79	0.98
ENSRNOG00000029945	Cuzd1	CUB and zona pellucida-like domains 1	2.25	-2.22	0.98
ENSRNOG00000062245	Ndufa10l1	NADH dehydrogenase (ubiquinone) 1 alpha subcomplex 10-like 1	1.47	-1.41	0.96
ENSRNOG00000042533	Accsl	1-aminocyclopropane-1-carboxylate synthase-like	2.84	-2.66	0.94
ENSRNOG00000002723	Sele	selectin E	1.14	-1.02	0.90
ENSRNOG00000030478	mt-Rnr1	s-rRNA	-1.28	1.08	0.84
ENSRNOG00000010261	Rnase10	ribonuclease A family member 10	-2.69	2.23	0.83
ENSRNOG00000043093	Ap1m2	adaptor-related protein complex 1, mu 2 subunit	-2.04	1.67	0.82
ENSRNOG00000015082	Nlrp10	NLR family, pyrin domain containing 10	2.42	-1.86	0.77
