# Supplementary material for: NMDA Receptors Regulate Oxidative Damage in Keratinocytes during Complex Regional Pain Syndrome in HaCaT Cells and Male Rats
Source: Antioxidants (Basel). 2024 Feb 18;13(2):244. doi: 10.3390/antiox13020244 (PMC10886417; doi:10.3390/antiox13020244)
Supplement: Supplementary file 1 [file antioxidants-13-00244-s001.zip › 1.Supplementary figures.zip!/Supplementary Figures.pdf]

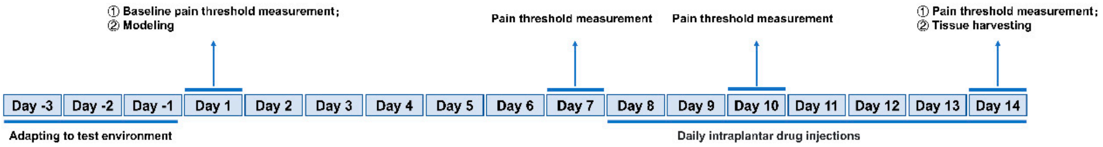

Supplementary Figure S1. Timeline of animal experiments.

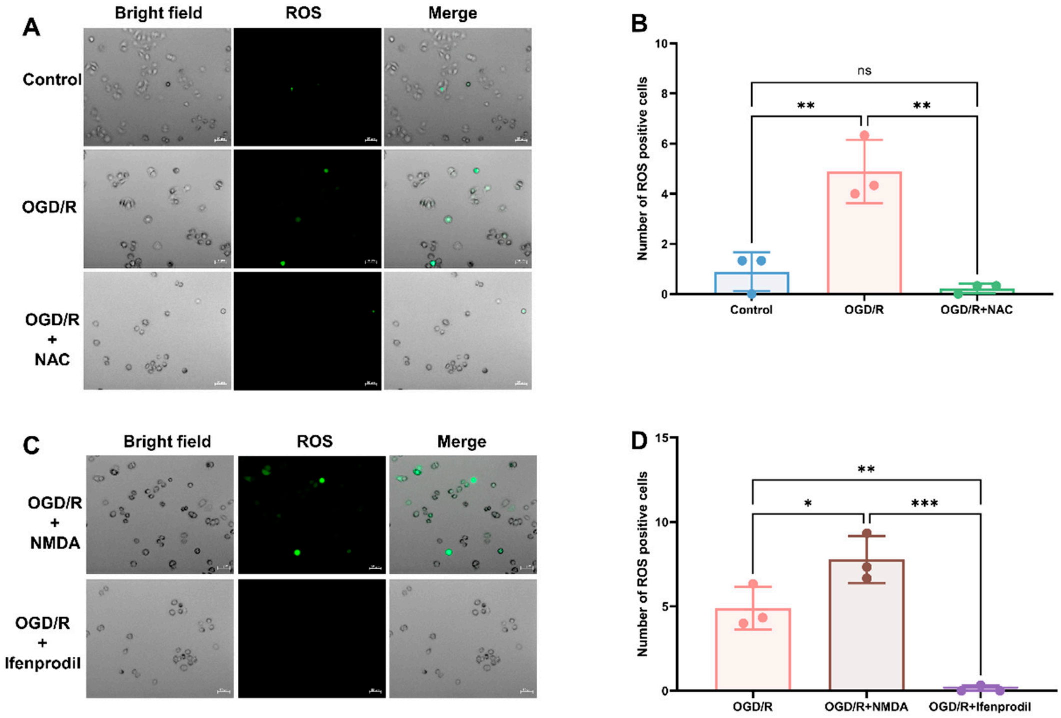

Supplementary Figure S2. ROS level in HaCaT cells after different treatments. (A) ROS staining indicated an increase in ROS-positive cells after OGD/R, which was reduced following NAC treatment. (B) Quantitative analysis of ROS positive cells in A. (C) ROS staining indicated that NR2B influences the number of ROS-positive cells after OGD/R. (D) Quantitative analysis of ROS-positive cells in C. ns, no significance; *, $p < 0.05$; **, $p < 0.01$; ***, $p < 0.005$. NAC, N-acetyl-L-cysteine; OGD/R, oxygen-glucose deprivation/reoxygenation; ROS, reactive oxygen species.

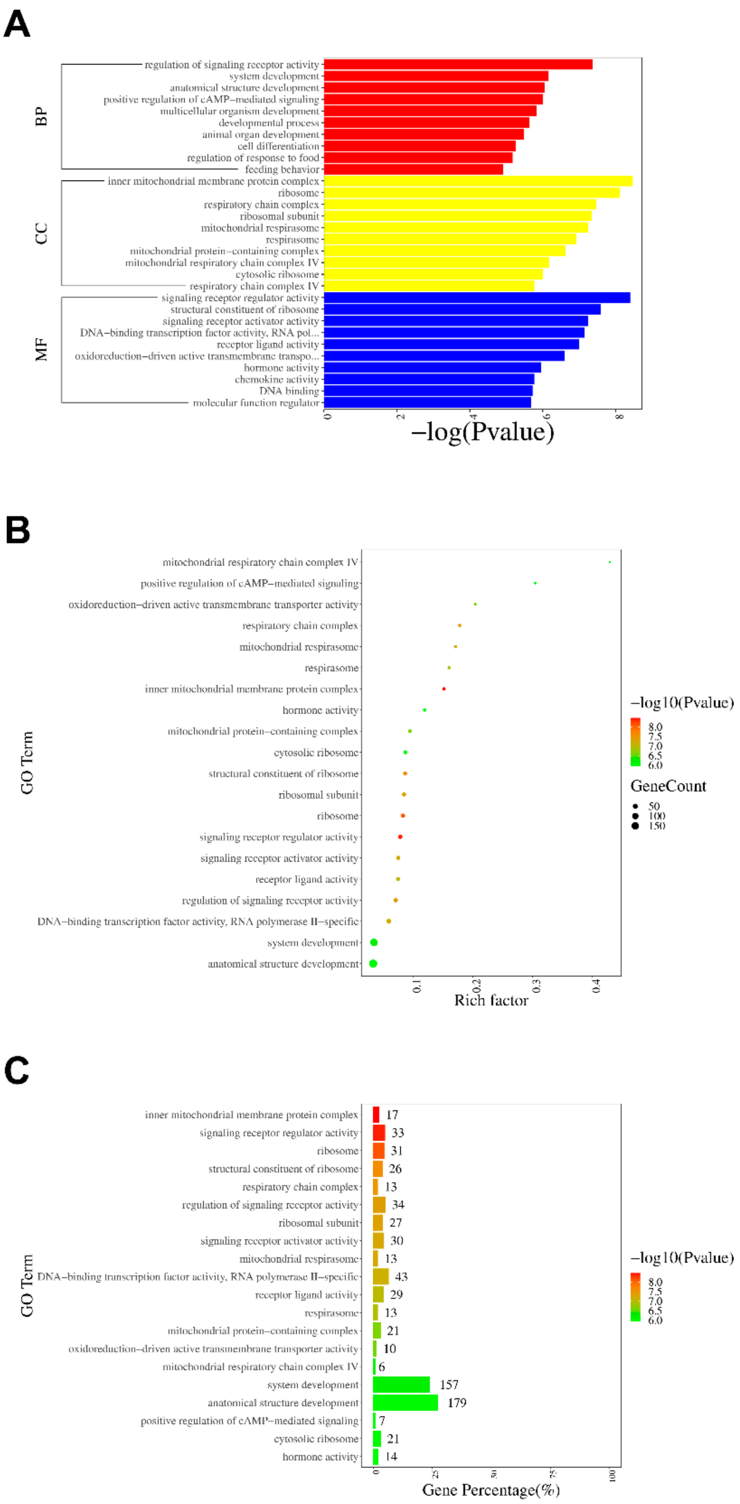

Supplementary Figure S3. GO pathway analysis of upregulated DEGs in DRGs from rats after intraplantar injection of ifenprodil. A: The bar plot shows the top 10 significantly up-regulated pathways related to BP, CC, and MF in DEGs obtained from CPIP-I vs CPIP. B: Bubble plot shows the top 20 most significantly up-regulated GO pathways related to BP, CC, and MF in DEGs obtained from CPIP-I vs CPIP. C: The bar chart shows the top 20 up-regulated pathways with the most enriched genes in DEGs obtained from CPIP-I vs CPIP. DRG, dorsal root ganglion; GO: gene ontology; BP: biological process; CC: cellular components; MF: molecular function; DEGs: differentially expressed genes; CPIP, chronic postischemia pain; I, ifenprodil.

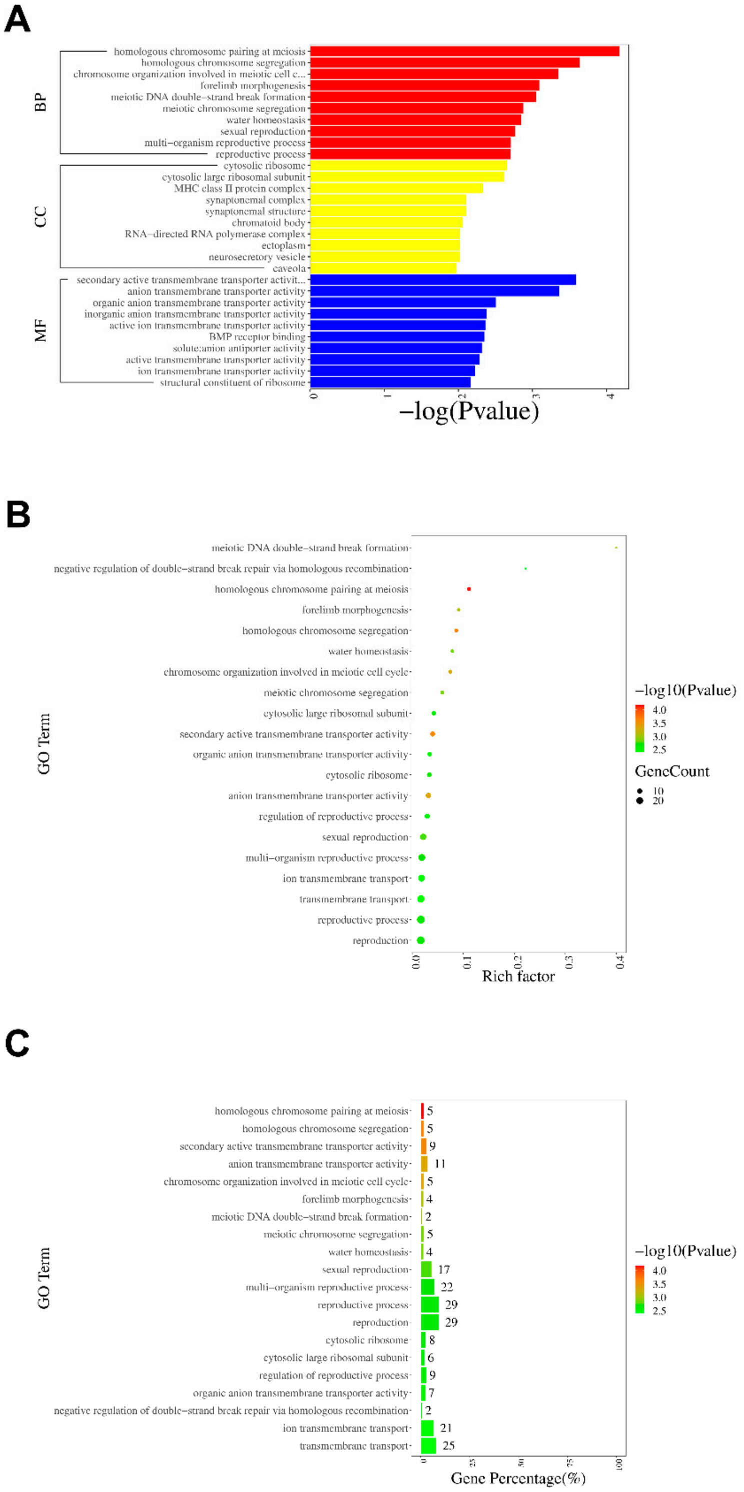

Supplementary Figure S4. GO pathway analysis of downregulated DEGs in DRGs from rats after intraplantar injection of ifenprodil. A: The bar plot shows the top 10 significantly downregulated pathways related to BP, CC, and MF in DEGs obtained from CPIP-I vs CPIP. B: Bubble plot shows the top 20 most significantly downregulated GO pathways related to BP, CC, and MF in DEGs obtained from CPIP-I vs CPIP. C: The bar chart shows the top 20 downregulated pathways with the most enriched genes in DEGs obtained from CPIP-I vs CPIP. DRG, dorsal root ganglion; GO: gene ontology; BP: biological process; CC: cellular components; MF: molecular function; DEGs: differentially expressed genes; CPIP, chronic postischemia pain; I, ifenprodil.

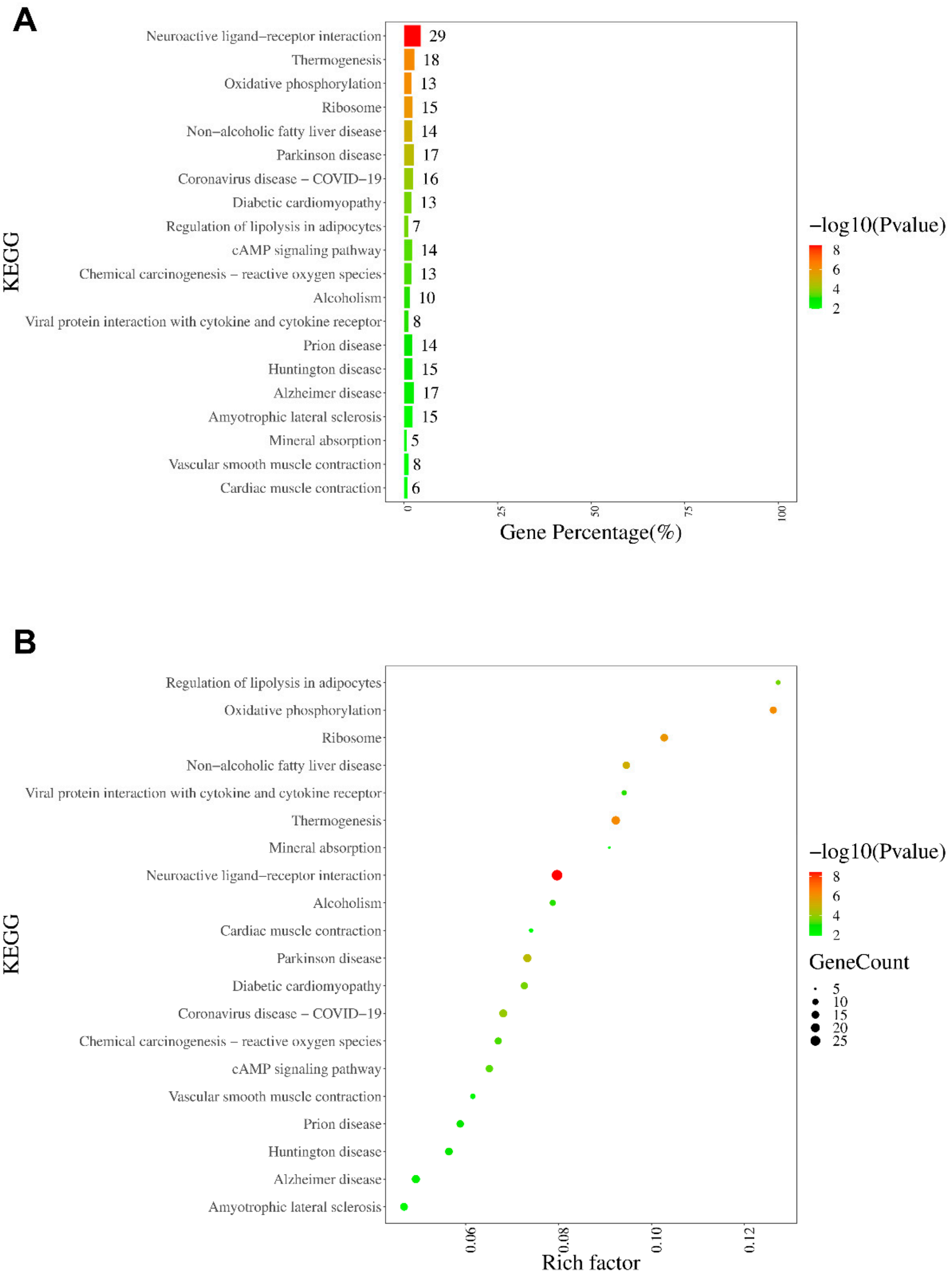

Supplementary Figure S5. KEGG pathway analysis of upregulated DEGs in DRGs from rats after intraplantar injection of ifenprodil. A: The bar chart shows the top 20 upregulated pathways with the most enriched genes in DEGs obtained from CPIP-I vs CPIP. B: Bubble plot shows the top 20 most significantly upregulated enriched KEGG pathways in DEGs obtained from CPIP-I vs CPIP. KEGG: kyoto encyclopedia of genes and genomes; DRG, dorsal root ganglion; DEGs: differentially expressed genes; CPIP, chronic postischemia pain; I, ifenprodil.

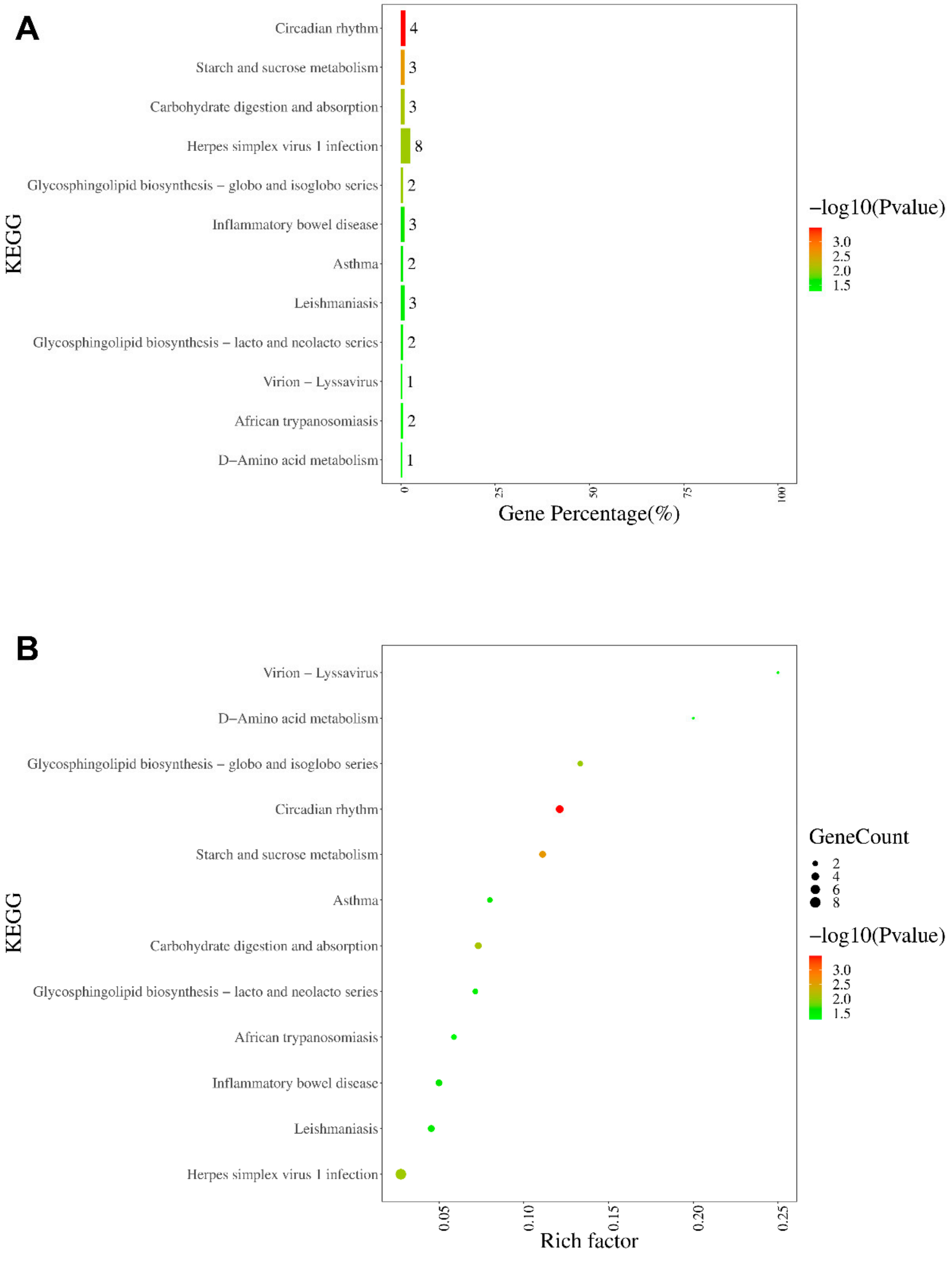

Supplementary Figure S6. KEGG pathway analysis of downregulated DEGs in DRGs from rats after intraplantar injection of ifenprodil. A: The bar chart shows the top 20 downregulated pathways with the most genes in DEGs obtained from CPIP-I vs CPIP. B: Bubble plot shows the top 20 most significantly downregulated KEGG pathways in DEGs obtained from CPIP-I vs CPIP. KEGG: kyoto encyclopedia of genes and genomes; DRG, dorsal root ganglion; DEGs: differentially expressed genes; CPIP, chronic postischemia pain; I, ifenprodil.
